# Optical control of the nuclear bile acid receptor FXR with a photohormone†
†Electronic supplementary information (ESI) available. See DOI: 10.1039/c9sc02911g


**DOI:** 10.1039/c9sc02911g

**Published:** 2019-11-19

**Authors:** Johannes Morstein, Julie B. Trads, Konstantin Hinnah, Sabine Willems, David M. Barber, Michael Trauner, Daniel Merk, Dirk Trauner

**Affiliations:** a Department of Chemistry , New York University , New York , New York 10003 , USA . Email: dirktrauner@nyu.edu; b Department of Chemistry , Center for Integrated Protein Science , Ludwig Maximilians University Munich , 81377 Munich , Germany; c Institute of Pharmaceutical Chemistry , Goethe-University Frankfurt , Max-von-Laue-Strasse 9 , 60438 Frankfurt , Germany; d Hans Popper Laboratory of Molecular Hepatology , Division of Gastroenterology and Hepatology , Department of Internal Medicine III , Medical University of Vienna , Waehringer Guertel 18-20 , 1090 Vienna , Austria

## Abstract

Herein, we report a photoswitchable modulator for a nuclear hormone receptor that exerts its hormonal effects in a light-dependent fashion. The azobenzene **AzoGW** enables optical control of the farnesoid X receptor (FXR), a key regulator of hepatic bile acid, lipid and glucose metabolism. **AzoGW** was derived from the synthetic agonist GW4064 through an azologization strategy and is a metabolically stable, highly selective photoswitchable FXR agonist in its dark-adapted form. Upon irradiation, the thermally bistable ‘photohormone’ becomes significantly less active. Optical control of FXR was demonstrated in a luminescence reporter gene assay and through light-dependent reversible transcription modulation of FXR target genes (*CYP7A1*, *Ostα*, *Ostβ*) in liver cells.

## Abstract 2

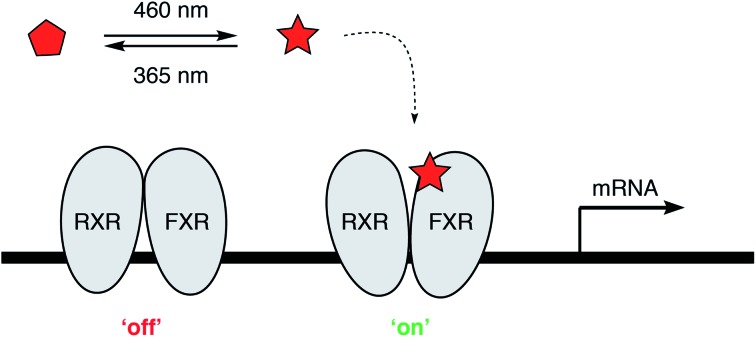
Herein, we report a photoswitchable modulator for a nuclear hormone receptor that exerts its hormonal effects in a light-dependent fashion.

## 

Nuclear hormone receptors (NHRs) are ligand-activated transcription factors which interact with DNA and regulate the expression levels of their target genes.^1^,^2^ As such, they are involved in a broad spectrum of biological processes, ranging from embryonic development, differentiation, homeostasis and metabolism to cell death (endocrine mode of control). A significant portion (*ca.* 16%) of all small molecule drugs act on this biological target family, which consists of 48 different receptors in humans.^3^ Endogenous ligands include steroid and thyroid hormones, retinoids, bile acids (BAs), fatty acids, and vitamin D.^4^

The farnesoid X receptor (FXR) is activated by BAs as endogenous ligands and serves as a central regulator of mammalian metabolism, that controls genes regulating the levels of BAs, lipids, cholesterol, and glucose.^5^,^6^ It is involved in various physiological and pathophysiological processes, including liver protection and regeneration, inflammation, and carcinogenesis. FXR acts on genes directly involved in BA metabolism and exhibits a number of rapid non-genomic effects, *e.g.* the control of glucose stimulated insulin secretion and glutamate transport.^7^

In recent years, photopharmacology has become a broadly applicable approach to manipulate and study biological systems.^8^–^10^ While ion channels, GPCRs, enzymes and many other biological targets have been successfully put under reversible optical control with small molecule photoswitches, NHRs have not yet been systematically addressed. Recently, we demonstrated that the azobenzene-containing retinoic acid receptor α (RARα) agonist Azo80 11 allows for optical control of RARα.^12^ We envisioned that photoswitchable NHR modulators, termed ‘photohormones’, could facilitate progress in the study of NHRs. Herein, we describe the development of a photoswitchable FXR agonist, termed **AzoGW**. This photohormone allows for optical control of FXR as demonstrated through a luminescent reporter gene assay and *via* transcriptional modulation in a liver cell model system.

**AzoGW** was derived from the potent FXR agonist GW4064. GW4064 is one of the most widely used FXR agonists with good selectivity over related NHRs.^13^ However, it has recently been shown to exhibit some off-target effects on GPCRs. GW4064 features a *trans*-stilbene moiety. Replacement of the stilbene with an azobenzene (‘azologization’)^12^,^14^ furnished the photoswitchable analog (Fig. 1A), which was synthesized in analogy to its parent compound (Fig. 1B). Diazotization of *m*-aminobenzoic acid and azo-coupling with phenol gave azobenzene **1**. A subsequent Fischer esterification afforded the corresponding methyl ester **2**. In parallel, 2,6-dichlorobenzaldoxime was chlorinated to yield chloro-oxime **3**. Treatment of **3** with 4-methyl-3-oxopentanoate under basic conditions then gave isoxazole **4**. DIBAL-H reduction yielded primary alcohol **5**, which was coupled with the azobenzene **2** under Mitsunobu conditions. Saponification of the resulting methyl ester then gave **AzoGW**.

**Fig. 1:**
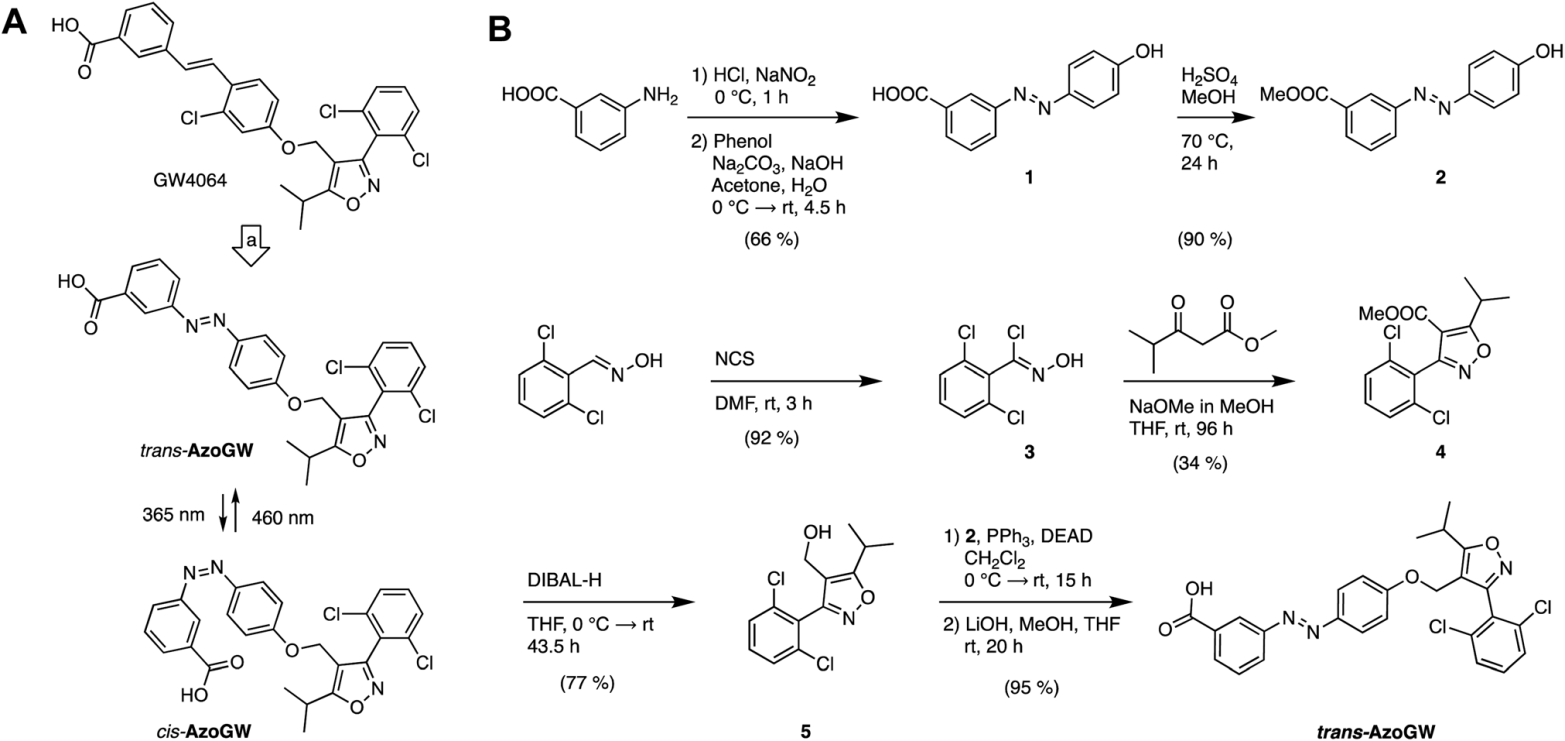
Design and synthesis of the photoswitchable FXR agonist **AzoGW**. (A) Design of **AzoGW** from the potent FXR agonist GW4064 *via* azologization. (B) Synthesis of **AzoGW**.

The photophysical properties of **AzoGW** were evaluated using UV-Vis spectroscopy (Fig. 2A–D). The UV-Vis spectra of **AzoGW** after illumination with *λ* = 460 nm (*trans*, blue) and *λ* = 365 nm (*cis*, gray) demonstrate wavelength-dependent switching as expected for a ‘classical’ azobenzene (Fig. 2A). Photoswitching could be repeated rapidly over multiple cycles (Fig. 2B), the amount of active photoswitch could be titrated with different wavelengths of light (‘color-dosing’, Fig. 2C), and no photobleaching, photodegradation, or fatigue was observed after numerous switching cycles (Fig. 2D). *cis*-**AzoGW** exhibits a thermal relaxation half-life time of 52 h at room temperature in physiological buffer (ESI Fig. S1†) and can thus be classified as a bistable photoswitch.

**Fig. 2:**
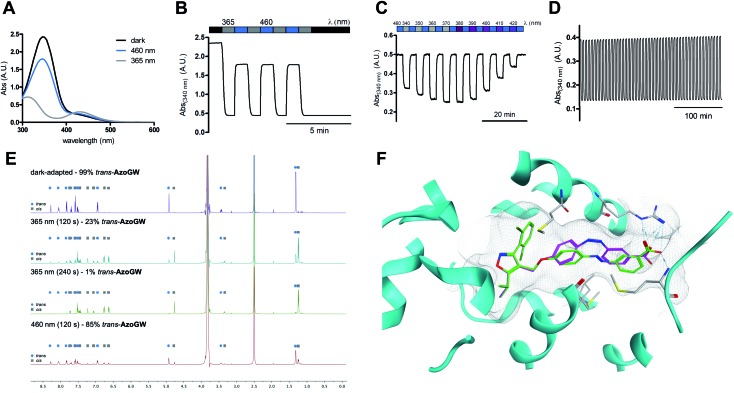
Photophysical evaluation and molecular docking of **AzoGW**. (A) The UV-Vis spectra of **AzoGW** in the dark-adapted (*trans*, black), 365 nm adapted (*cis*, gray) and 460 nm adapted (*trans*, blue) photostationary states. (B) Reversible cycling between photoisomers with alternating illumination at the two distinct wavelengths 365 nm and 460 nm, indicating the fast kinetics of the isomerization. (C) Reversible cycling between photoisomers with alternating illumination at varying wavelengths (between 340 nm and 420 nm). (D) **AzoGW** was repeatedly switched between its *cis*- and *trans*-state for 50 cycles. (E) ^1^H-NMR-studies of **AzoGW** in the dark-adapted, 365 nm adapted and 460 nm adapted photostationary states in DMSO-d_6_/D_2_O. (F) Molecular docking of *trans*-**AzoGW** (green) and *cis*-**AzoGW** (purple) to the FXR ligand binding site (PDB-ID: ; 3DCT 15).

In some cases, the metabolic stability of azobenzenes is compromised by reductive cleavage by bacteria or glutathione (GSH) reduction.^16^ To assess the metabolic stability of **AzoGW**, we assayed its stability in the presence of the bacterial enzyme azoreductase in *E. coli* (ESI Fig. S5†). We found that **AzoGW** exhibits excellent stability whilst methyl red is degraded over time. Additionally, we evaluated the stability of **AzoGW** in the presence of GSH (ESI Fig. S6†). Although GSH enhances the thermal relaxation of *cis*-**AzoGW**, we did not observe notable degradation, supporting that **AzoGW** is metabolically stable which makes it potentially useful for applications in cultured cells or *in vivo*.

We further investigated the binding mode of **AzoGW** by molecular docking to a co-crystal of GW4064 and FXR (PDB-ID: ; 3DCT 15). The docking pose of *trans*-**AzoGW** matches the crystallized binding mode of GW4064 (ESI Fig. S7†). The *cis*-isomer of **AzoGW** deviates considerably from the binding pose of GW4064 and *trans*-**AzoGW** and the carboxylic acid group is less close to engaging residues (Fig. 2E). These results suggest reduced potency of the *cis*-isomer in keeping with the predictions of our chemoinformatic azologization study.^12^

To explore if **AzoGW** allows for light-dependent activation of FXR, we carried out a luminescent reporter gene assay for human FXR, where the ligand-binding dependent activation of FXR is coupled to the expression of the reporter gene firefly luciferase. We used varying concentrations of GW4064 and **AzoGW** (Fig. 3A). A Cell DISCO system was employed to achieve switching of *trans*-**AzoGW** to *cis*-**AzoGW** (370 nm, 75 ms pulse every 15 s).^17^ We found that *trans*-**AzoGW** (dark, EC_50_ = 1.1 × 10^–6^ M) is approximately 100-fold more potent than the less active *cis*-isomer of **AzoGW** (UV, EC_50_ = 9.5 × 10^–5^ M). These results demonstrate that good optical control of FXR activation could be obtained with this tool at low micromolar concentrations. As a control experiment, we showed that light does not affect transcription levels (Fig. 3B). Moreover, the FXR antagonist DY 268 (2 μM) inhibits the effects of *trans*-**AzoGW** (367 nM) demonstrating target-specificity in our assay. A rescue experiment demonstrated reversibility of the reported effects. To this end, **AzoGW** (367 nM) was added to cells as 365 nm-adapted *cis*-**AzoGW** and after 5 min the cells were illuminated with 460 nm light for 2 min to reactivate **AzoGW**. Similar levels of transcription were observed compared to the experiment with dark-adapted *trans*-**AzoGW** (Fig. 3B). In a hybrid reporter gene assay for the nuclear hormone receptors PPARα/γ/δ, RXRα/β/γ, LXRα/β, RARα/β/γ, FXR, VDR, and CAR, we verified that *trans*-**AzoGW** selectively activates FXR over other related receptors (Fig. 3C).

**Fig. 3:**
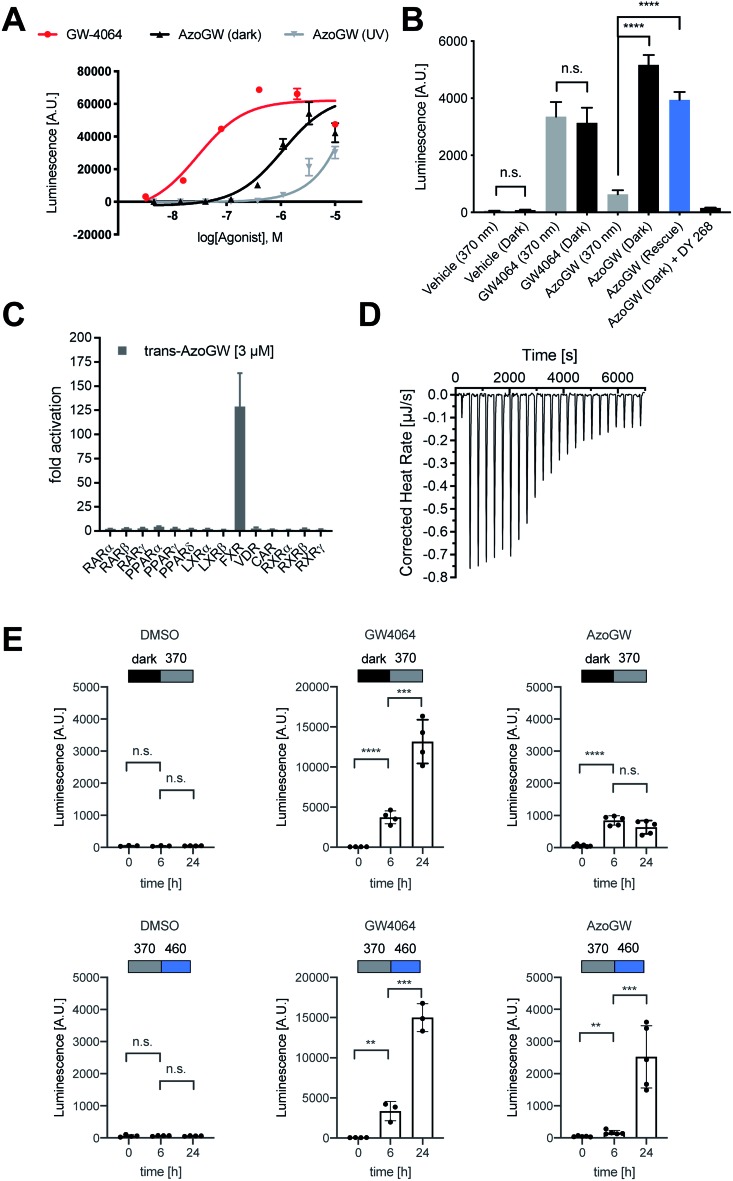
Pharmacological evaluation of **AzoGW**. (A) Dose responses of GW4064, *trans*-**AzoGW** and *cis*-**AzoGW** in a luminescent reporter cell line after 22 h. Samples were run in duplicate and in two independent experiments. Error bars represent mean ± SD (B) control and rescue (reversibility) experiments using GW4064 (41 nM), **AzoGW** (367 nM), and DY 268 (2 μM). Samples were run at least in triplicate. Error bars represent SEM ***p < 0.001, n.s., not significant, Student's *t*-test. (C) Selectivity panel for *trans*-**AzoGW** (3 μM) over several related nuclear hormone receptors. Samples were run in duplicate and in four independent experiments. Error bars represent SEM. (D) Isothermal titration calorimetry (ITC) experiment with recombinant FXR LBD and **AzoGW** (dark-adapted, 15 μM). *K*_D_ = 433 nm. (E) Bidirectional switching experiment with **AzoGW** in luminescent reporter cell line using GW4064 (0.5 μM) and **AzoGW** (2 μM). To switch from dark to 370 nm cells were removed from the dark after 6 hours, irradiated with 365 nm for 3 minutes and subsequently irradiated with the Cell DISCO (370 nm) system for 75 ms every 15 s. To switch from 365 nm cells were removed from the Cell DISCO (370 nm) system after 6 hours, irradiated at 465 nm for 3 minutes and incubated in the dark until 24 h.

Next, we analyzed the capacity of **AzoGW** for the optical control of FXR-dependent gene expression in untransfected liver cells (HepG2). To this end, we analyzed expression levels of *CYP7A1* as well as *Ostα*/*β* mRNA. *CYP7A1* encodes a monooxygenase that accounts for the rate-limiting step in bile acid biosynthesis. *Ostα* and *Ostβ* encode bile acids transporters (Fig. 4A). As expected, treatment of HepG2 cells with GW4064 resulted in a robust suppression of *CYP7A1*, whereas a strong upregulation of *Ostα* and *Ostβ* was observed (Fig. 4C–E). Expression levels of FXR remained unaffected (Fig. 4B). We also saw no differences in the light-excluded (dark) and irradiated samples (365 nm) with GW4064, confirming that light at the intensities used in our experiments does not mediate changes in FXR-dependent transcription levels.

**Fig. 4:**
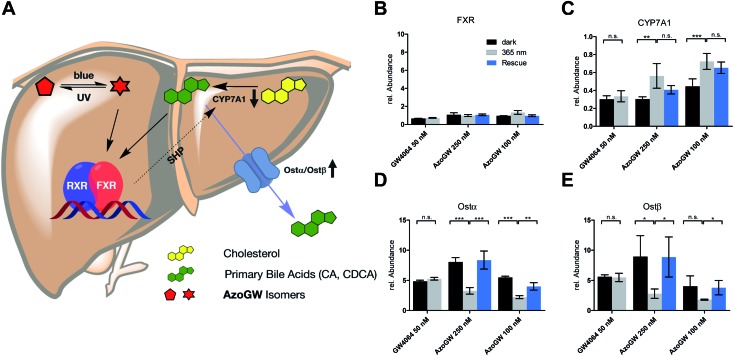
(A) Role of FXR in physiology: Agonist induced activation of FXR, either by its endogenous ligands (BA) or synthetic ligands (GW4064, **AzoGW**), leads to an up-or downregulation of its target genes. FXR indirectly suppresses *CYP7A1* which catalyzes the rate determining step in BA biosynthesis from cholesterol. Also, FXR promotes expression levels of the BA export pumps *Ostα* and *Ostβ*. (B–E) Effects of GW4064 and **AzoGW** on FXR. Depicted are the mRNA expression levels of FXR, *CYP7A1*, *Ostα* and *Ostβ*, relative to the vehicle control (1% DMSO) after incubation for 24 h in the dark (black) or under illumination with *λ* = 365 nm (gray) and *λ* = 460 nm (blue) respectively. Samples were run in three independent experiments. Error bars represent SEM ***p < 0.001, **p < 0.01, *p < 0.1, n.s., not significant, Student's *t*-test.

Next, we investigated whether **AzoGW** could put FXR dependent gene expression under optical control. Indeed, incubation of HepG2 cells with different concentrations of **AzoGW** showed light-dependent suppression of *CYP7A1* and upregulation of *Ostα* and *Ostβ* (Fig. 4C–E). As expected, *trans*-**AzoGW** is a more potent agonist of FXR, leading to a stronger modulation of FXR target genes. Relative abundance of FXR did not change significantly. This supports that the observed expression level modulations exclusively originate in differences in the effect of *trans*-**AzoGW** and *cis*-**AzoGW** on FXR. A rescue experiment was conducted to assess the reversibility of the inhibitory and promoting effect of **AzoGW** (Fig. 4, blue bars) concluding that illumination with blue light (*λ* = 460 nm) significantly restores the activity of *trans*-**AzoGW** (referred to as the dark-adapted state).

In summary, we report the development of a photohormone for the nuclear hormone receptor FXR. **AzoGW** exhibits excellent photo- and metabolic stability and allows for photocontrol of FXR. Most importantly, FXR-dependent transcription was brought under optical control in an untransfected liver cell line, demonstrating the potential of this tool in cellular systems. Our study underscored that optogenetic and photopharmacological technologies can be applied to nuclear receptors, which include thyroid hormone receptors (TRs), peroxisome proliferator-activated receptors (PPARs), and estrogen receptors (ERs). In addition, to azologization strategies, many NHRs could potentially be addressed through the design of photoswitchable amphiphilic (lipid-like) agonists.^18^–^20^ Photohormones could enable precise genomic regulation in complex signaling networks and rapid control of non-genomic NHR effects. As such, they could lead to new insights into NHR physiology and find therapeutic applications using endoscopic/endoluminal light delivery.^21^,^22^

## Conflicts of interest

M. T. served as a speaker and/or consultant for Albireo, Boehringer Ingelheim, BiomX, Falk, Gilead, Intercept, Novartis, Phenex, Regulus and Shire, and received travel support from Falk, Gilead, and Intercept, as well as grants/research support from Albireo, Cymabay, Falk, Gilead and Intercept. He is also co-inventor of patents on the medical use of 24-norursodeoxycholic acid.

## Supplementary Material

Supplementary information
