# Supplementary material for: Optical control of the nuclear bile acid receptor FXR with a photohormone
Source: Chem Sci. 2019 Nov 19;11(2):429–34. doi: 10.1039/c9sc02911g (PMC7067245; doi:10.1039/c9sc02911g)

Electronic Supplementary Material (ESI) for Chemical Science.
This journal is © The Royal Society of Chemistry 2019

Supporting Information

Optical Control of the Nuclear Bile Acid Receptor FXR with a Photohormone

Johannes Morstein[a], Julie B. Trads[b], Konstantin Hinnah[a], Sabine Willems[c], David M. Barber[b], Michael Trauner[d], Daniel Merk[c], and Dirk Trauner*[a]

[a]Department of Chemistry, New York University, 100 Washington Square East, New York, NY 10003-6699, USA.

[b]Department of Chemistry and Center for Integrated Protein Science, Ludwig Maximilians University Munich, Butenandtstraße 5-13, 81377 Munich, Germany.

[c]Institute of Pharmaceutical Chemistry, Goethe-University Frankfurt, Max-von-Laue-Strasse 9, 60438 Frankfurt, Germany.

[d]Hans Popper Laboratory of Molecular Hepatology, Division of Gastroenterology and Hepatology, Department of Internal Medicine III, Medical University of Vienna, Waehringer Guertel 18-20, A-1090 Vienna, Austria.

Table of Contents

Reagents and Instrumentation 3

Photophysical Characterization 4

Half-life determination 5

Computational Methods 7

Cell culture 8

FXR Reporter Gene Assay 9

Selectivity Assay 10

Isothermal Titration Calorimetry 12

Treatment of HepG2 cells for Gene Expression 14

RNA Isolation and Reverse Transcription 15

Real-Time Quantitative PCR 16

Cell DISCO Control Experiment 17

MTT viability assay 19

Azoreductase Assay 21

GSH Assay 23

Docking of *trans*-AzoGW 25

GW4064 dose response 26

Synthesis of AzoGW 28

- 3-((4-Hydroxyphenyl)diazenyl)benzoic acid (1)[5] 28
- Methyl 3-((4-hydroxyphenyl)diazenyl)benzoate (2) 29
- (*Z*)-2,6-Dichloro-*N*-hydroxybenzimidoyl chloride (3)[6] 30
- Methyl 3-(2,6-dichlorophenyl)-5-isopropylisoxazole-4-carboxylate (4)[6] 31
- (3-(2,6-Dichlorophenyl)-5-isopropylisoxazol-4-yl)methanol (5)[6] 32
- 3-((4-((3-(2,6-dichlorophenyl)-5-isopropylisoxazol-4-yl)methoxy)phenyl)diazenyl)benzoic acid (AzoGW) 33

References 34

^{1}H and ^{13}C NMR Spectra 35

- 3-((4-Hydroxyphenyl)diazenyl)benzoic acid (1) 35
- Methyl 3-((4-hydroxyphenyl)diazenyl)benzoate (2) 36
- (*Z*)-2,6-Dichloro-*N*-hydroxybenzimidoyl chloride (3) 37
- Methyl 3-(2,6-dichlorophenyl)-5-isopropylisoxazole-4-carboxylate (4) 38
- (3-(2,6-Dichlorophenyl)-5-isopropylisoxazol-4-yl)methanol (5) 39
- 3-((4-((3-(2,6-dichlorophenyl)-5-isopropylisoxazol-4-yl)methoxy)phenyl)diazenyl)benzoic acid (AzoGW) 40

Reagents and Instrumentation

All reagents and solvents were purchased from commercial sources (Sigma-Aldrich, TCI Europe N.V., Strem Chemicals, etc.) and were used without further purification. Solvents were obtained from Fisher Scientific. HepG2 cells were obtained from ATCC. Reactions were monitored by TLC on precoated, Merck Silica gel 60 F_{254} glass backed plates and the chromatograms were first visualized by UV irradiation at λ = 254 nm. Flash silica gel chromatography was performed using silica gel (SiO_2, particle size 40-63 μm) purchased from SiliCycle. NMR spectra were measured on a BRUKER Avance III HD 400 (equipped with a CryoProbe™). Multiplicities in the following experimental procedures are abbreviated as follows: s = singlet, d = doublet, t = triplet, q = quartet, m = multiplet. Proton chemical shifts are expressed in parts per million (ppm, δ scale) and are referenced to the residual protium in the NMR solvent ($CDCl_3$ = 7.26; MeOD: δ = 3.31, $(CD_3)_2SO$: δ = 2.50, C_6D_6: δ = 7.16). Carbon chemical shifts are expressed in ppm (δ scale) and are referenced to the carbon resonance of the NMR solvent ($CDCl_3$ = 77.16; MeOD: δ = 49.00, $(CD_3)_2SO$: δ = 39.52, C_6D_6: δ = 128.06). NOTE: Due to the *trans*/*cis* isomerization of some compounds containing an azobenzene functionality, more signals were observed in the ^1H and ^{13}C spectra than would be expected for the pure *trans*-isomer. IR spectra were recorded on a PerkinElmer Spectrum BX II FT-IR instrument equipped with an ATR unit. The measured wave numbers are reported in cm^{-1}. All high-resolution mass spectra (HRMS) were recorded by the LMU mass spectrometry service. HRMS were recorded on MAT 95 (EI) and MAT 90 (ESI) spectrometers from Thermo Finnigan GmbH. The method used is reported in the experimental section.

RNA concentration measurements were performed on a FLuOStar Omega well plate reader (BMG Labteach). The reverse transcription was performed using a C1000 Thermal Cycler (BioRad) and the real-time quantitative PCR using a LightCycler 480 (Roche).

The LED light sources were obtained from Amazon (λ= 365 nm and λ= 460 nm) and LEDSupply (LuxStrip II LED bar, λ= 660 nm) respectively. The cell 'Disco' system is in-house made (described previously).[1] 24 x 5mm LEDs 370nm (XSL-370-5E) from Roithner Lasertechnik were used for the Cell DISCO experiments.

Photophysical Characterization

UV-Vis spectra were recorded using a Varian Cary 50 Bio UV-Visible Spectrophotometer with Hellma SUPRASIL precision cuvettes (10 mm light path). Switching was achieved using 365 nm or 460 nm LED light sources. The LEDs were pointed directly onto the top of the sample cuvette. An initial spectrum was recorded (dark-adapted state, black) and then again following illumination at λ = 365 nm for 30 s (*cis*-adapted state, gray). A third spectrum was recorded after irradiation at λ = 470 nm for 30 s (*trans*-adapted state, blue).

In order to obtain the reversible *trans* ←→ *cis* spectrum, absorption at λ = 340 nm was constantly measured whilst alternating illumination at λ = 365 nm or λ = 460 nm allowed for rapid isomerization of **AzoGW** (125 µM in 10 % DMSO and 90 % PBS). Like before, the light source was directly pointed onto the top of the sample cuvette. Several switching cycles were performed. Subsequently, we performed a second reversible *trans* ←→ *cis* spectrum with multiple cycles, in order to further investigate the photostability of **AzoGW**. A 89 North Optoscan monochromator was used as lightsource with a glass fiber cable to point the light onto the cuvette. A total number of 50 switching cycles has been performed, resulting in a total illumination time of 300 mins.

For the action spectrum, the same instrumental setup was used, comprising the monochromator and the glass fiber cable, the latter which directly pointed onto the top of the sample cuvette. **AzoGW** (25 µM in 10 % DMSO and 90 % PBS) was illuminated with the indicated wavelengths for 3 min and absorption at λ = 340 nm was recorded for 90 mins in total.

Half-life determination

Determination of the half-life time of *cis*-**AzoGW** was achieved using a Varian Cary 50 Bio UV-Visible Spectrophotometer with Hellma SUPRASIL precision cuvettes (10 mm light path). Switching was achieved using a 365 nm light source. The absorption of 50 µM **AzoGW** in 10 % DMSO and 90 % PBS at λ= 340 nm was measured every 15 seconds at 20 °C for 15 h in total.

Initially, the absorption of *trans*-**AzoGW** (dark-adapted state) was measured for 5 mins. Then, illumination at λ= 365 nm for 1 min provided *cis*-**AzoGW**. Finally, the natural isomerization rate at room temperature in the dark was analyzed.

Analyzing the acquired data provided a half-life time of 52.4 h, assuming a one-phase decay.

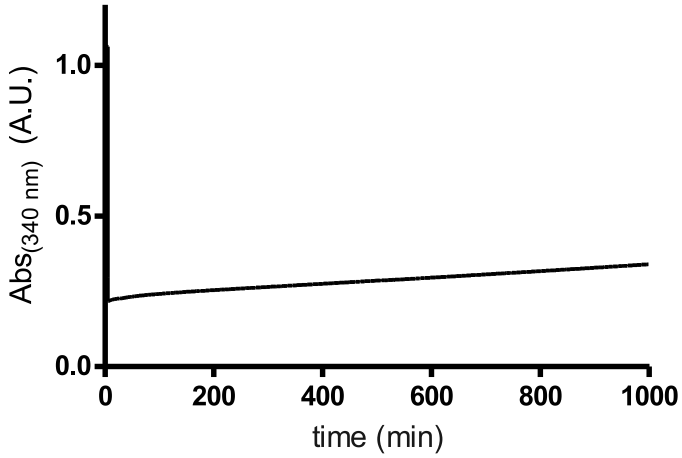

Figure SI 1. Thermal Relaxation of **AzoGW**.

Computational Methods

General

Calculations were conducted in Molecular Operating Environment (MOE, version 2018.0101, Chemical Computing Group Inc. Montreal, QC, Canada) using default settings for each tool/function unless stated otherwise.

Molecular Docking

Docking was performed using X-ray structure of FXR ligand binding domain (PDB code 3DCT) in complex with GW4064. Protonation state of the complex was adjusted using the MOE QuickPrep tool with default settings. Redocking of the crystallized ligand resulted in a binding pose with RMSD 0.1248 relative to the crystallized binding mode. Compounds *trans*-AzoGW and *cis*-AzoGW were prepared using the MOE Wash tool: Protonation state dominant at pH 7; Coordinates Rebuild 3D; Preserved Existing Chirality. Docking was performed using following settings from MOE Dock tool: Receptor: Receptor + Solvent; Site: Ligand Atoms; Placement: Triangle Matcher; Score: London dG; Poses: 100; Refinement: Rigid receptor; Refinement Score: GBVI/WSA dG; Poses: 10. The top-ranked binding-mode was used.

Cell culture

HepG2 cells, a human hepatoma cell line, were maintained in T25 flasks with DMEM containing 10 % fetal bovine serum and 1 % penicillin/streptomycin at 37 °C in a humidified 5 % CO_2 atmosphere. For passaging, cells were grown to near confluency, then washed once with warm PBS, detached with 0.25 % trypsin and finally transferred to a new flask every two days following standard procedures (only HepG2 cells until passage 20 were used)

The cell line was tested negative for contaminations and mycoplasma using the Cell Culture Contamination Detection Kit (Thermo Fischer Scientific).

FXR Reporter Gene Assay

A cell-based human FXR (*NR1H4*) driven luciferase reporter assay from INDIGO Bioscience (State College, PA) was adapted and used for the biological evaluation of **AzoGW**. In brief, a 5 mM stock solution of GW4064 or **AzoGW** was diluted with the provided cell screening medium to a final concentration of 100 µM. A 5-fold dilution series was prepared using this initial concentration and cell screening medium. The dilutions were added to reporter cells in white-bottom 96-well plates. For ***trans*-AzoGW**, dilutions were added in the dark and cells were incubated for 22 h in the dark. For ***cis*-AzoGW**, dilutions were irradiated at 365 nm for 3 minutes and cells were incubated for 22 h in the presence of a 370 nm LED Cell Disco with light pulses for 75 ms/15 s. To minimize variations in the dilutions, the same dilutions were used for both experiments before and after irradiation. After 22 h medium was ejected and the supplied luciferase detection reagents were added and quantified using a BMG Labtech FLUOstar Omega plate reader. Samples were run in duplicates and from two independent experiments.

Selectivity Assay

Hybrid Reporter Gene Assays for PPARα/γ/δ, RXRα/β/γ, LXRα/β, RARα/β/γ, FXR, VDR, CAR.

Plasmids. The Gal4-fusion receptor plasmids pFA-CMV-hPPARα-LBD, pFA-CMV-hPPARγ-LBD, pFA-CMV-hPPARδ-LBD, pFA-CMV-hLXRα-LBD, pFA-CMV-hLXRβ-LBD, pFA-CMV-hRXRα-LBD, pFA-CMV-hRXRβ-LBD, pFA-CMV-hRXRγ-LBD, pFA-CMV-hRARα-LBD, pFA-CMV-hRARβ-LBD, pFA-CMV-hRARγ-LBD, pFA-CMV-hFXR-LBD, pFA-CMV-hVDR-LBD and pFA-CMV-hCAR-LBD coding for the hinge region and ligand binding domain (LBD) of the canonical isoform of the respective nuclear receptor have been reported previously. pFR-Luc (Stratagene) was used as the reporter plasmid and pRL-SV40 (Promega) for normalization of transfection efficiency and cell growth.

Assay Procedure

HEK293T cells were cultured in Dulbecco's modified Eagle's medium (DMEM), high glucose with 10% fetal calf serum (FCS), sodium pyruvate (1 mM), penicillin (100 U/mL), and streptomycin (100µg/mL) at 37°C and 5% CO_2. Twenty-four hours before transfection, HEK293T cells were seeded in 96-well plates (3×10^4 cells/well). Before transfection, medium was changed to Opti-MEM without supplements. Transient transfection was carried out using Lipofectamine LTX reagent (Invitrogen) according to the manufacturer's protocol with pFR-Luc (Stratagene), pRL-SV40 (Promega), and the corresponding Gal4-fusion nuclear receptor plasmid. Five hours after transfection, medium was changed to Opti-MEM supplemented with penicillin (100 U/mL), streptomycin (100µg/mL), and additionally containing 0.1 % DMSO and the respective reference agonist (1 µM) or *trans*-**AzoGW** (3 µM) or 0.1 % DMSO alone as untreated control. Each concentration was tested in duplicates and each experiment was repeated independently four times. After overnight (14–16 h) incubation with the test compounds, the cells were assayed for luciferase activity using the Dual-Glo Luciferase Assay System (Promega) according to the manufacturer's protocol. Luminescence was measured with an Infinite M200 luminometer (Tecan Deutschland GmbH). Normalization of transfection efficiency and cell growth was done by division of firefly luciferase data by Renilla luciferase data and multiplying the value by 1000 resulting in relative light units (RLU). Fold activation was obtained by dividing the mean RLU of *trans*-**AzoGW** at 3 µM by the mean RLU of the untreated control. All hybrid assays were validated with the respective reference agonists (PPARα: GW7647; PPARγ: pioglitazone; PPARδ:

L165,041; RXRα/β/γ: bexarotene; RARα/β/γ: tretinoin; LXRα/β: T09021317; FXR: GW4064; CAR: CITCO; VDR: calcitriol) which yielded EC_{50} values in agreement with the literature.

Isothermal Titration Calorimetry

ITC was conducted on a TA Instruments Affinity ITC (TA Instruments, New Castle, Delaware, USA) using recombinant FXR LBD protein (expressed and purified as described previously[2,3]) dissolved in buffer at pH 8.3 containing 10 mM Tris, 100 mM NaCl, 5 mM DTT and 1% DMSO. **AzoGW** was dissolved to a final concentration of 15 µM in the same buffer, placed into the ITC cell (172 µL) and inversely titrated with FXR LBD protein (100 µM). The titration was performed at a temperature of 25 °C with a stirring rate of 75 rpm and 23 injections. The first injection had a reduced volume of 0.5 µL, followed by 22 injections of 2.5 µL. An interval of 300 s was maintained between individual injections. ITC raw data were analysed using NanoAnalyze software package (version 3.7.5). An independent binding model was used to fit the reaction enthalpy (ΔH), binding affinity constant (K_d), and stoichiometry (n). Free energy change (ΔG) was calculated from the equation $\Delta G = -RT \ln K$ and the entropy (ΔS) was calculated from $\Delta G = \Delta H - T\Delta S$.

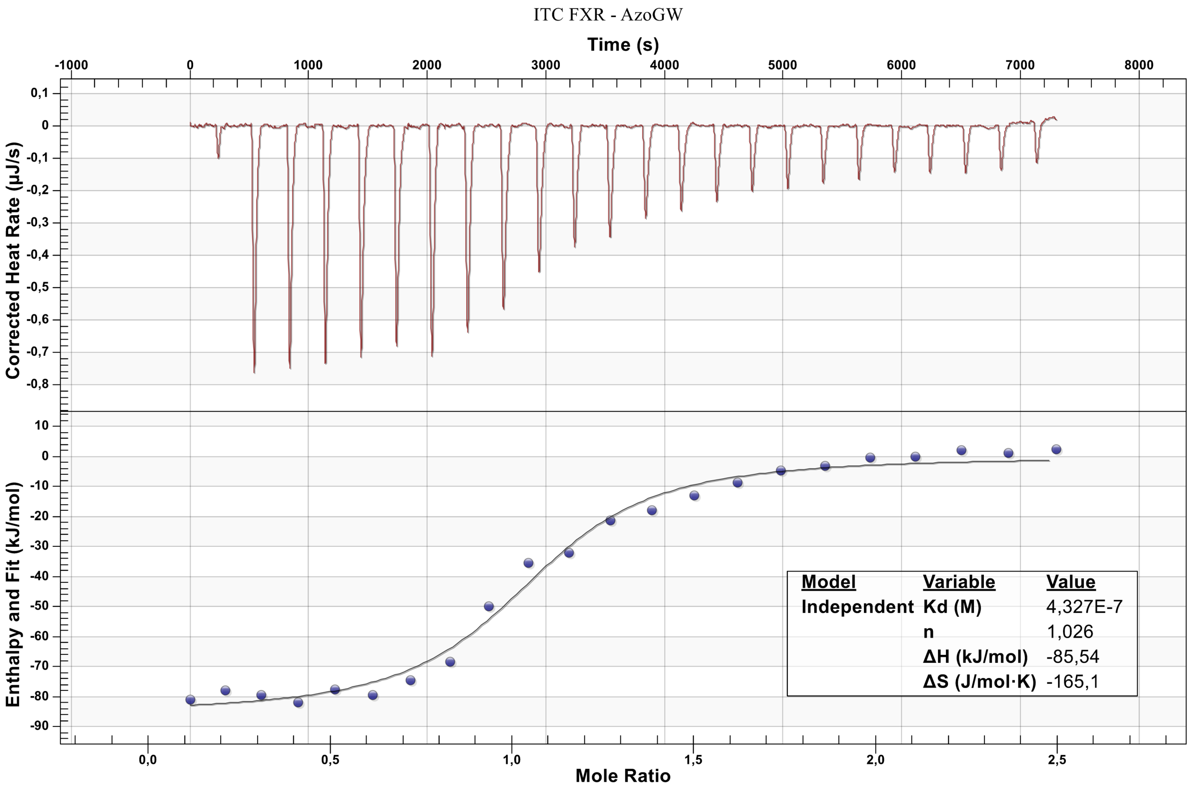

Figure SI 2. Isothermal Titration Calorimetry (ITC) experiment with FXR LBD and **AzoGW**.

Treatment of HepG2 cells for Gene Expression

The HepG2 cells were grown to near confluency and then seeded in 6-well plates at a density of 10^6 cells/well in 3 mL fresh medium (including 10 % FBS and 1 % PS) ~24 h prior to treatment. Cells were washed with warm PBS and then incubated with 1 % DMSO, 50 nM GW4064, 250 nM and 100 nM **AzoGW** in 3 mL medium without phenol red and FBS + 1 % PS for 24 h (compounds were diluted from 100x stock solutions in DMSO).

The whole experiment was performed in a completely dark room with a red LED bar (λ= 660 nm) as the only source of light. Cells were incubated at 37 °C in 5 % CO_2 atmosphere in light-proof boxes in the dark ('dark') or with pulsed illumination using a custom-made "Disco" LED lighting system ('365 nm') after pre-irradiation of the compounds as described previously.[1]

In addition, a rescue experiment has been performed. In doing so, the **AzoGW** solutions were pre-irradiated with λ= 365 nm for 2 mins, applied to the cells and then illuminated with λ= 460 nm for 2 mins. The cells were also incubated at 37 °C in 5 % CO_2 atmosphere in a light-proof box.

RNA Isolation and Reverse Transcription

Total RNA from HepG2 cells was isolated using the RNeasy Mini Kit (Qiagen), following the provided protocol. RNA concentrations were obtained in duplicates using the FluoStar Omega well-plate reader by BMG LabTech.

Reverse transcription was performed according the manufacturer's instructions (High capacity cDNA Reverse Transcription Kit by Applied Biosystems). Briefly, 10 µL ddH_2O containing 1000 ng RNA and 10 µL 2x RT-PCR Master Mix (containing RT Buffer, RT random Primers, dNTP Mix and MultiScribe Reverse Transcriptase) were thoroughly mixed and reversely transcribed using the C1000 Thermal Cycler by BioRad.

Table SI 1: Temperature program for reverse transcription of total RNA from HepG2 cells.

Temperature / °C	Time / min
25	10
37	120
85	5
4	∞

Real-Time Quantitative PCR

In order to determine the expression levels of specific genes a real-time quantitative PCR was performed. Shortly, 18 µL of a freshly prepared qPCR Master Mix (containing the PowerUp^{TM} SYBR^{TM} Green Master Mix (Applied Biosystems), forward and reverse primers (0.5 µM final concentration) and ddH_2O) were transferred to every well on a white 96-well plate. Finally, 2 ng of cDNA were added to each well prior to amplification. The 96-well plate was sealed and analyzed with the LightCycler 480 by Roche, running the following program. Every sample was run in technical as well as biological triplicates.

Table SI 2: Temperature program for reverse transcription of total RNA from HepG2 cells.

Step	Temperature / °C	Time / min	# of cycles
1	95	10	1
2	95	0.25	40
3	60	60	

Table SI 3: Nucleobase sequences for forward and reverse Primers for real-time quantitative PCR of the indicated genes.

Gene	Forward	Reverse
Actin	GACGACATGGAGAAAAATCTG	ATGATCTGGGTCATCTTCTC
FXR	TCTCCTGGGTCGCCTGACT	ACTGCACGTCCCAGATTTCAC
CYP7A1	CCATAAGGTGTTGTGCCACGGAAA	GCCCAAATGCCTTCGCAGAAG
Ostα	TTCCAGGTTCTCCTCATCCTGAC	AATTCATCACTTGAGACCTGGTTTT
Ostβ	GGTGGTCATTATAAGCATGGTCCT	CTGGTGGCTGCATCGTTTCT

Cell DISCO Control Experiment

Due to potentially altered thermal relaxation in a cellular environment (temperature, GSH, etc.) – an additional control experiment was performed, in order to evaluate, if illumination with the Cell Disco system is sufficient for keeping the **AzoGW** in its *cis*-conformation. In order to do so, the absorption spectra for two separate solutions of **AzoGW** (50 µM in 10 % DMSO and 90 % PBS) on two distinct 96-well plates was performed initially (run in triplicates, using the FluoStar Omega well plate reader). After that, the plates were transferred to light-proof boxes in the incubator at 37 °C in 5 % CO_2 atmosphere. The Cell Disco system was employed to afford illumination of one plate, whereas the control plate was kept in the dark.

As depicted in figure SI 2, incubation in the dark at 37 °C and 5 % CO_2 almost entirely affords the *trans*-**AzoGW**, whereas the absorption spectrum of 50 µM **AzoGW** illuminated with the Cell Disco system (λ = 365 nm) shows no difference in the *cis*-**AzoGW** and after 24 h spectrum. This indicates that illumination is sufficient to compensate the natural isomerization rate and to keep the photoswitchable agonist in the *cis*-state.

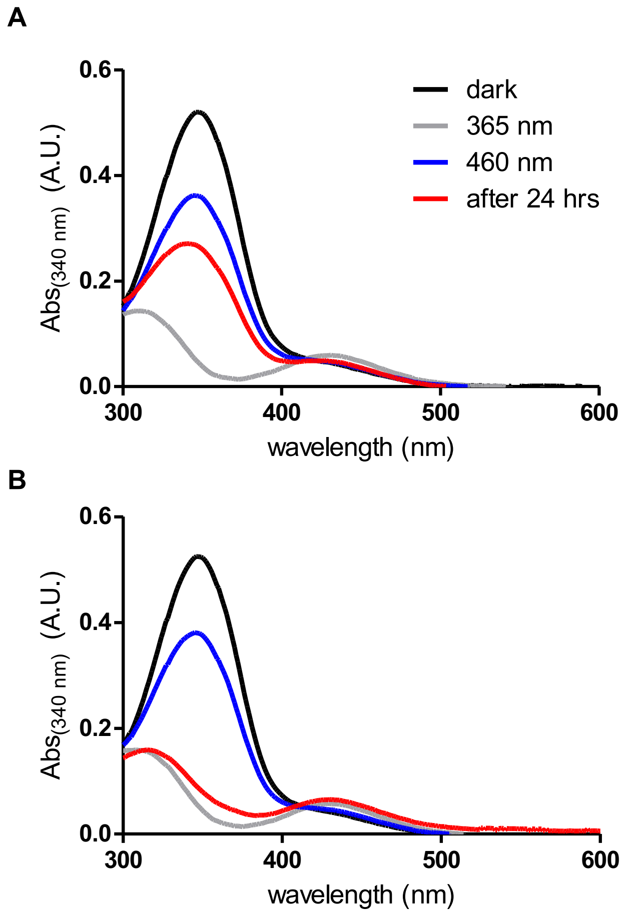

Figure SI 3. Absoprtion spectra of 50 µM **AzoGW** before and after incubation in the dark (A) and illumination with the cell disco (B) at 37 °C and 5 % CO_2 for 24 h.

MTT viability assay

The MTT viability assesses the metabolic activity of cells. It is based on the reduction of the yellow tetrazolium salt MTT to purple formazan by a dehydrogenase found in the mitochondria of living cells. Organic solvents, such as DMSO dissolve the purple crystals and form a purple solution, who's absorption at λ = 550 nm can be determined colorimetrically. The reduction potential and the color of the solution along with it are proportional to the number of living cells. Therefore, an MTT viability assay presents a popular method to assess the toxicity of a compound for cells.

For the MTT viability assay, HepG2 cells were removed by trypsinization from an exponentially-phase maintenance culture (in DMEM and 10 % FBS and 1 % PS at 37 °C and 5 % CO_2). Cells were seeded on a poly-lysine pre-coated 96-well plate in 100 µL culture medium at a density of 5k – 10k cells per well. Cells attached overnight in the incubator at 37 °C and then 100 µL medium, medium with drug (10 nM – 50 µM **AzoGW** in 1 % DMSO) or medium with vehicle only were added to the appropriate wells. After 24 h treatment in light-proof boxes in the incubator at 37 °C, cells were washed twice with phenol red free medium, following incubation with 0.5 mg/mL MTT for 4 h at 37 °C (therefore dilute a 12 mM MTT stock solution made from 5 mg MTT in 1 mL sterile-filtrated PBS in medium accordingly). After treatment, MTT was replaced by 100 µL DMSO to dissolve the formazan crystals. After 5 mins at 37 °C mix each well thoroughly with a multi-channel pipette before reading the absorption at 550 nm.

Cell viability was calculate using the following formula

$$\%\, cell\ viability = \frac{absorbance\ of\ sample}{absorbance\ of\ control} * 100$$

in which the wells incubated with medium only represent the controls. Figure SI 3 shows the results of the MTT viability assay for different concentrations of **AzoGW** (dark-adapted state). Data shown represent the average and SD of five experiments.

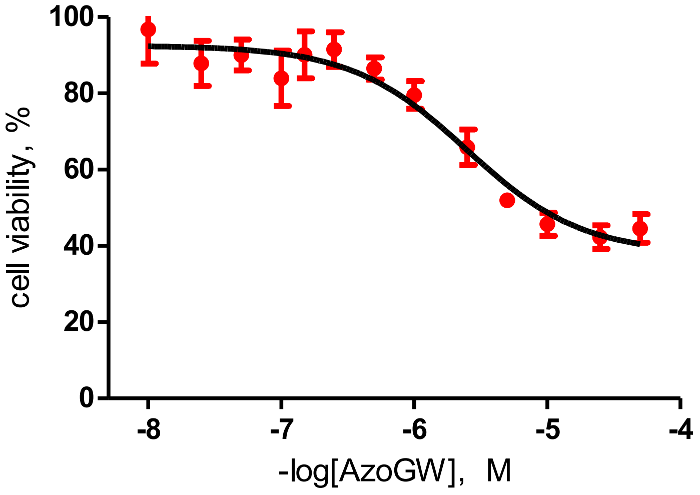

Figure SI 4. MTT viability assay for **AzoGW** in HepG2 cells. Cells were incubated with different concentrations of **AzoGW** (dark-adapted state), ranging from 10 nM to 50 μM (in 1 % DMSO).

Azoreductase Assay

The azoreductase assay tests for the cleavage of azobenze derivatives into aniline products by the enzyme azoreductase. *E.coli* bacteria serve as a model system in this regard since they are known to carry azoreductase.

The assay was performed by transforming a plasmid containing an ampicillin resistance gene into competent *E.coli* bacteria and outgrowing a single colony in 50 mL LB medium + ampicillin to OD = 0.6. Next, 5 mL of the bacteria LB medium suspension were transferred into three culture tubes and 5 µL of DMSO, methyl red (50 mM in DMSO) or **AzoGW** (50 mM in DMSO, dark-adapted) were added to the respective tubes, resulting in a final concentration of 50 µM. The compounds were inoculated at 37 °C and 180 RPM and a 200 µL aliquot was taken immediately before and 1, 5, 10, 15. 25, 45 and 90 min after the beginning of the inoculation.

Aliquots were centrifuged at 12.000 RPM at 4 °C for 1 min and stored on ice immediately. 100 µL of the supernatant of each tube was transferred to a well on a 96-well plate and the absorbance at the respective λ_{max} (430 nm for methyl red and 340 nm for **AzoGW**) was read.

In parallel, 250 µL of each sample were plated on an ampicillin containing agar plate and incubated at 37 °C and 5 % CO_2 for 16 h. Finally, pictures were taken from the methyl red, **AzoGW** and DMSO control plate.

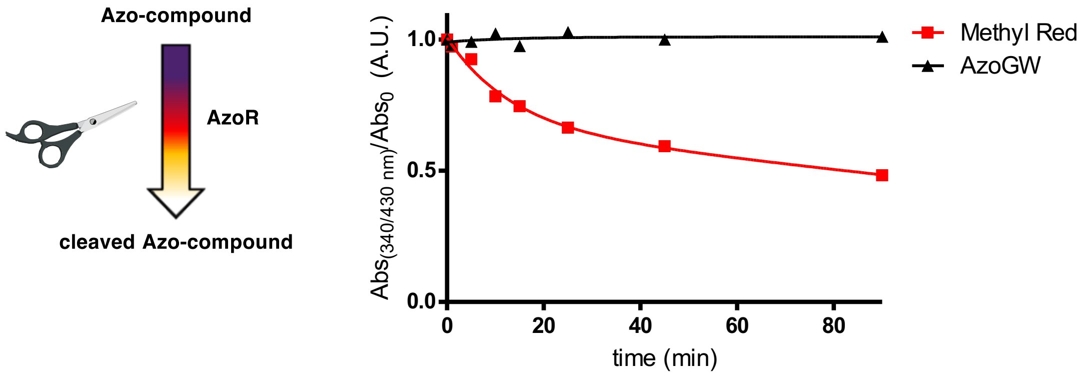

Figure SI 5. Azoreductase Assay. Absorption of **AzoGW** at 340 nm and methyl red (red) at 430 nm over the course of time (intensities were normalized to achieve comparability).

GSH Assay

AzoGW (50 µM in 1 % DMSO and PBS) was incubated at 37 °C in the presence of fully reduced glutathione (2.5 mM in PBS) and TCEP (1 mM in PBS) on a 96-well plate (total volume 100 µL). Absorbance was read every minute for 2 h (λ = 340 nm). While one plate was excluded from light (accounting for the *trans*-**AzoGW**), the second plate was pre-illuminated with λ = 365 nm for three minutes.

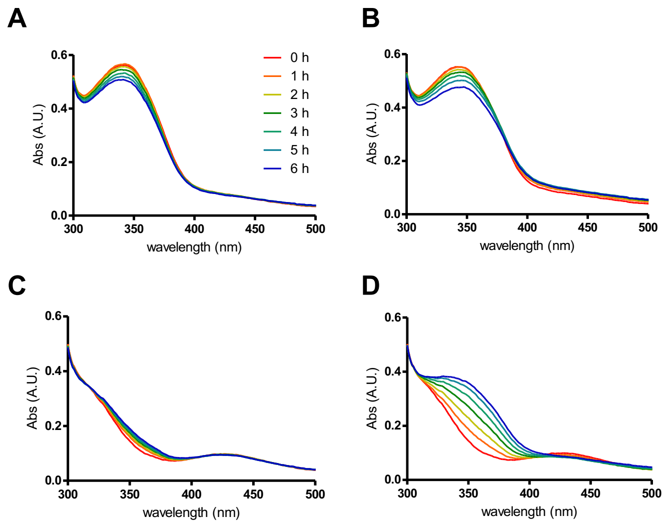

Figure SI 6. (A-D) Glutathione reduction assay. (A,B) Absorption spectra of *trans*-AzoGW at different times after incubation with DMSO (A) or 2.5 mM GSH and 1 mM TCEP (B). (C,D) Absorption of *cis*-AzoGW at different times after incubation with DMSO (C) or 2.5 mM GSH and 1 mM TCEP (D).

Docking of *trans*-AzoGW

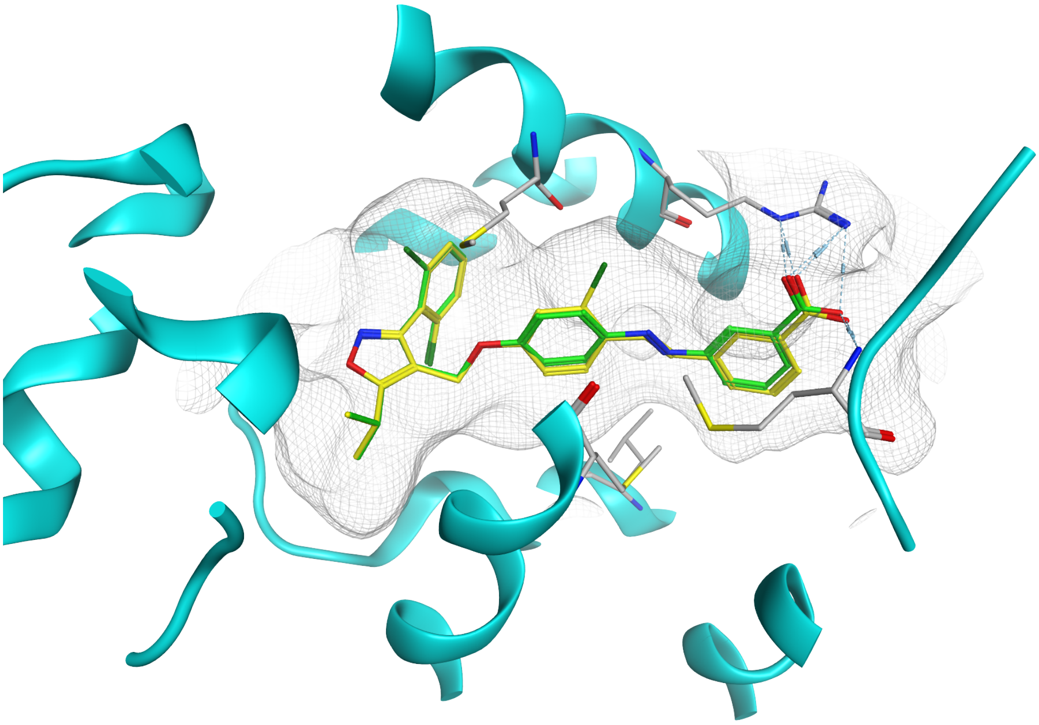

Figure SI 7. Molecular docking of *trans*-**AzoGW** (green) and GW4064 (yellow) to the FXR (PDB-ID: 3DCT[4]) ligand binding site.

GW4064 dose response

HepG2 cells were grown to near confluency and then seeded in a 6-well plate at a density of 10^6 cells/well in 3 mL fresh medium (including 10 % FBS and 1 % PS) ~24 h prior to treatment. Cells were washed with warm PBS and then incubated with different concentrations of GW4064 (1 µM, 250 nM, 50 nM, 10 nM and 1 nM) in 3 mL medium without phenol red and FBS + 1 % PS for 24 h (compounds were diluted from 100x stock solutions in DMSO). RNA isolation, RT and qPCR were performed as described previously.

Figure SI 7 shows the expression levels of the target genes *FXR, CYP7A1* and *Ost*α in HepG2 cells 24 h after incubation with the indicated concentration of GW4064. Whereas stable expression levels of *FXR* were observed throughout the whole concentration range, a dose-dependent suppression of *CYP7A1* and dose-dependent upregulation of *Ost*α was observed.

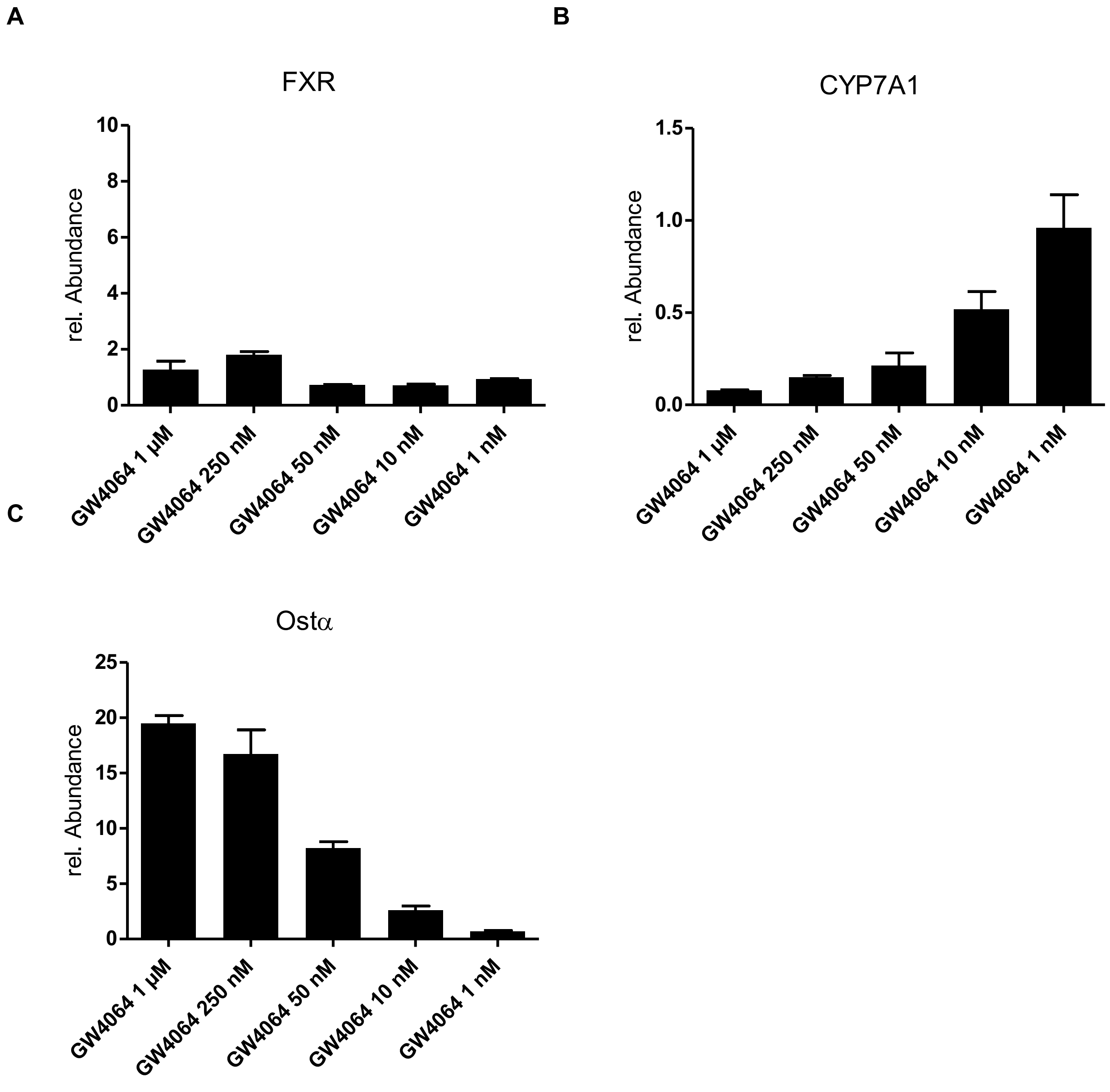

Figure SI 8. Expression levels of target genes ((A) *FXR*, (B) *CYP7A1*, (C) *Ost*α of FXR in HepG2 cells 24 h after treatment with the indicated concentration of GW4064.

Synthesis of AzoGW

3-((4-Hydroxyphenyl)diazenyl)benzoic acid (1)[5]

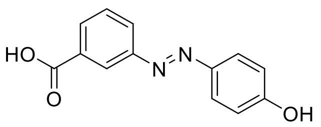

3-Aminobenzoic acid (295 mg, 2.15 mmol, 1.2 eq.) was dissolved in a mixture of acetone and water (1:1, 20 ml) and cooled to 0 °C before conc. HCl (1.15 ml) was added dropwise. After 5 min sodium nitrite (173 mg, 2.51 mmol, 1.4 eq) in water (1.4 ml) was added dropwise. After 1 h at 0 °C the mixture was cannulated into a mixture of phenol (168 mg, 1.79 mmol, 1 eq.), Na_2CO_3 (379 mg, 3.58 mmol, 2 eq.), and NaOH (286 mg, 7.16 mmol, 4 eq.) in acetone and water (1:1, 20 ml). The reaction mixture was stirred at rt. for 4.5 h before the volume was reduced *in vacuo* and HCl (1M, aq. 10 mL) was added. The aqueous solution was cooled in the freezer. The formed precipitate was isolated by filtration and washed with water x2, CH_2Cl_2 x2 to yield **1** as a red solid (287 mg, 1.19 mmol, 66%).

Rf (CH_2Cl_2/MeOH 95:5) = 0.23

^{1}H NMR (400 MHz, MeOD) δ 8.45 (s, 1H), 8.15 – 8.03 (m, 2H), 7.87 (d, *J* = 8.3, 2H), 7.63 (t, *J* = 7.7, 1H), 6.93 (d, *J* = 8.3, 2H).

^{13}C NMR (100 MHz, MeOD) δ 169.2, 162.6, 154.2, 147.4, 133.1, 132.1, 130.4, 127.7, 126.3, 124.3, 116.8.

IR (cm^{-1}): ṽ = 2821, 2565, 1678, 1589, 1307, 1223, 833.

HRMS (ESI): calcd for $C_{13}H_{11}N_2O_3^+$ $(M+H)^+$: 243.0764; found: 243.07638.

mp (uncorr.): 222.8-224.3 °C (lit.[1] 217 °C)

^{1}H-NMR and ^{13}C-NMR spectra are in accordance with literature values.[5]

Methyl 3-((4-hydroxyphenyl)diazenyl)benzoate (2)

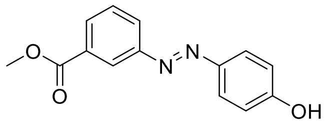

3-((4-Hydroxyphenyl)diazenyl)benzoic acid (100 mg, 0.413 mmol, 1 eq.) was dissolved in MeOH (2ml) and three drops of conc. H_2SO_4 was added. The reaction mixture was heated to 70 °C in a sealed tube. After 24 h it was poured onto ice and extracted with CH_2Cl_2 x3. The combined organic phases were washed with saturated $NaHCO_3$ and brine. The solvent was dried over Na_2SO_4 and evaporated *in vacuo* to yield the product without further purification as orange solid (95.3 mg, 371 mmol, 90 %).

Rf (iHex/EtOAc 1:1) = 0.73/0.87 *cis/trans*

^{1}H NMR (400 MHz, $CDCl_3$) δ 8.53 (s, 1H), 8.11 (d, *J* = 7.8, 1H), 8.06 (d, *J* = 7.8, 1H), 7.95 (d, *J* = 8.7, 1H), 7.90 (d, *J* = 8.7, 1H), 7.58 (t, *J* = 7.8, 1H), 7.03 (d, *J* = 8.7, 1H), 6.97 (d, *J* = 8.7, 1H), 5.78 (br. s, 1H), 3.97 (s, 3H).

^{13}C NMR (100 MHz, $CDCl_3$) δ 166.9, 162.5, 159.0, 152.8, 147.0, 131.3, 129.3, 126.9, 125.4, 125.2, 123.8, 116.0, 114.4, 55.8.

IR (cm^{-1}): $\tilde{\nu}$ = 3435, 2959, 2544, 1696, 1601, 1500, 1443, 1302, 1263, 1128, 979, 848, 769.

HRMS (ESI): calcd for $C_{14}H_{13}N_2O_3^+$ $(M+H)^+$: 257.09179; found: 257.09209.

mp (uncorr.): 173.2-174.3 °C

(*Z*)-2,6-Dichloro-*N*-hydroxybenzimidoyl chloride (3)[6]

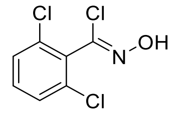

2,6-Dichlorobenzaldehyde oxime (1.00 g, 5.26 mmol, 1 eq.) was dissolved in DMF and *N*-chlorosuccinimide (703 mg, 5.26 mmol, 1 eq.) was added while the mixture was kept at rt. in a water bath. After 3 h TLC analysis showed full consumption of starting material and the reaction mixture was distributed between Et_2O and water. The organic phase was washed with water x3 and brine. It was dried over Na_2SO_4 and concentrated *in vacuo* to yield **3** as a colorless solid without further purification (1.09 g, 4.85 mmol, 92%).

Rf (iHex/EtOAc 9:1) = 0.50

^{1}H NMR (400 MHz, $CDCl_3$) δ 8.05 (s, 1H), 7.43 – 7.29 (m, 3H).

^{13}C NMR (100 MHz, $CDCl_3$) δ 135.5, 134.2, 131.8, 128.9, 128.3.

IR (cm^{-1}): ṽ =3333, 2300, 1560, 1430, 1349, 1194, 930, 777.

HRMS (EI): calcd for $C_7H_4Cl_3NO^+$ $(M)^{+\cdot}$: 222.93530; found: 273222.9360.

mp (uncorr.): 79.4-81.0 °C (lit.[3] 92-93 °C)

^{1}H-NMR spectrum is in accordance with literature values. [6]

Methyl 3-(2,6-dichlorophenyl)-5-isopropylisoxazole-4-carboxylate (4)[6]

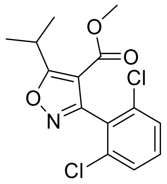

NaH 60% dispersion in mineral oil (31.6 mg, 0.790 mmol, 1.14 eq.) was dissolved in MeOH and heated to 50 °C for 10 min. to form a 0.5 M solution of NaOMe in MeOH. To this solution was added a solution of methyl 4-methyl-3-oxopentanoate (100 mg, 694 mmol, 1 eq.) in THF (0.75 ml) and the reaction mixture was stirred at rt. for 20 min. before a solution of 2,6-dichloro-*N*-hydroxybenzimidoyl chloride (156 mg, 0.694 mmol, 1 eq.) in THF (0.25 ml) was added. After 4 days the reaction mixture was concentrated *in vacuo* and the residue was dissolved in Et_2O. The organic phase was washed with water, brine, and dried over Na_2SO_4. The solvent was concentrated *in vacuo* and the residue purified by flash column chromatography (iHex/EtOAc 95:5 - 9:1) to yield **4** as a colorless powder (73.7 mg, 0.235 mmol, 34 %).

Rf(iHex/EtOAc 9:1) = 0.41

^{1}H NMR (400 MHz, $CDCl_3$) δ 7.43 – 7.30 (m, 3H), 3.86 (hept, *J* = 7.0, 1H), 3.66 (s, 3H), 1.43 (d, *J* = 7.0, 6H).

^{13}C NMR (100 MHz, $CDCl_3$) δ 183.3, 161.7, 158.7, 135.4, 131.1, 128.4, 127.8, 107.5, 51.8, 27.9, 20.3.

IR (cm^{-1}): ṽ = 2976, 1715, 1597, 1433, 1341, 1294, 1057, 783.

HRMS (ESI): calcd for $C_{14}H_{14}NO_3^+$ $(M+H)^+$: 314.0426; found: 314.03472.

mp (uncorr.): 85.7-86.1 °C

^{1}H-NMR spectrum is in accordance with literature values.[6]

(3-(2,6-Dichlorophenyl)-5-isopropylisoxazol-4-yl)methanol (5)[6]

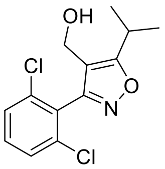

Methyl 3-(2,6-dichlorophenyl)-5-isopropylisoxazole-4-carboxylate (64.0 mg, 0.204 mmol, 1 eq.) was dissolved in THF (0.46 ml) and diisobutylaluminum hydride in toluene (1 M, 0.433 ml, 2.13 eq.) was added dropwise at 0 °C after which the reaction was allowed to reach rt. over several hours. There was not full consumption of starting material after 42 h and more diisobutylaluminum hydride in toluene (1 M, 0.433 ml, 2.13 eq.) was added at 0 °C. After 1.5 h the reaction was quenched by the addition of saturated potassium sodium tartrate at 0 °C and the solution was stirred 1 h. The solution was diluted with water and extracted with EtOAc. The organic phase was washed with water and brine. The solvent was dried over Na_2SO_4 and concentrated *in vacuo*. The residue was purified by flash column chromatography (iHex/EtOAc 8:2) to yield **5** as a colorless solid (44.8 mg, 0.157 mmol, 77%).

Rf (iHex/EtOAc 8:2) = 0.13

^{1}H NMR (400 MHz, $CDCl_3$) δ 7.45 – 7.31 (m, 3H), 4.32 (s, 2H), 3.33 (hept, *J* = 7.0, 1H), 1.58 (br. s, 1H), 1.41 (d, *J* = 7.0, 6H).

^{13}C NMR (100 MHz, $CDCl_3$) δ 176.4, 159.2, 135.7, 131.4, 128.3, 128.1, 112.7, 53.7, 27.0, 21.0.

IR (cm^{-1}): $\tilde{\nu}$ = 3412, 2973, 1618, 1560, 1430, 1194, 1018, 792.

HRMS (ESI): calcd for $C_{13}H_{14}Cl_2NO_2^+$ $(M+H)^+$: 286.03935; found: 286.03966.

mp (uncorr.): 111.9-112.7 °C

^{1}H-NMR spectrum is in accordance with literature values.[6]

3-((4-((3-(2,6-dichlorophenyl)-5-isopropylisoxazol-4-yl)methoxy)phenyl)diazenyl)benzoic acid (AzoGW)

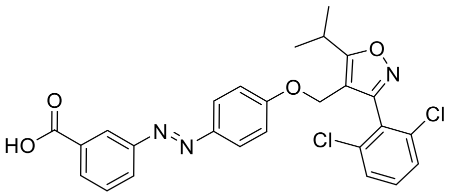

(3-(2,6-Dichlorophenyl)-5-isopropylisoxazol-4-yl)methanol (140 mg, 0.489 mmol, 1 eq.), methyl 3-((4-hydroxyphenyl)diazenyl)benzoate (125 mg, 0.489 mmol, 1 eq.), and triphenylphosphine (128 mg, 0.489 mmol, 1 eq.) were dissolved in CH_2Cl_2 (1.5 ml) and diisopropyl azodicarboxylate (98.9 mg, 0.489 mg, 1 eq.) was added dropwise. After 15 h the solvent was removed *in vacuo* and the residue was purified by flash column chromatography (iHex/EtOAc 98:2) to yield an orange oil. This oil was dissolved in a mixture of LiOH (1 M aq., 4 ml), MeOH (2 ml), and THF (4 ml). After 20 h the reaction mixture was acidified using HCl (10% aq.) and extracted with EtOAc x2. The combined organic phases were washed with brine and dried over Na_2SO_4. The solvent was concentrated *in vacuo* and purified by flash column chromatography (CH_2Cl_2/MeOH 95:5) to yield **AzoGW** as orange solid (106 mg, 0.208 mmol, 94%).

Rf (CH_2Cl_2/MeOH 95:5) = 0.25

^{1}H NMR (400 MHz, MeOD) E_{iso}: δ 8.47 (s, 1H), 8.10 (d, *J* = 7.7, 1H), 7.96 (d, *J* = 7.7, 1H), 7.87 – 7.80 (m, 2H), 7.59 – 7.38 (m, 4H), 6.97 – 6.90 (m, 2H), 4.92 (s, 2H), 3.46 (hept, *J* = 7.0, 1H), 1.43 (d, *J* = 7.0, 6H). Z_{iso}: δ 7.61 – 7.39 (m, 5H), 7.32 (t, *J* = 7.8, 1H), 6.86 (d, *J* = 7.8, 1H), 6.83 – 6.78 (m, 2H), 6.67 – 6.60 (m, 2H), 4.77 (s, 2H), 3.37 (hept, *J* = 7.0, 1H), 1.37 (d, *J* = 7.0, 3H).

^{13}C NMR (100 MHz, $CDCl_3$): δ 176.4, 160.9, 159.2, 152.5, 147.1, 135.3, 131.7, 131.0, 128.5, 128.0, 127.4, 124.8, 124.3, 123.3, 123.1, 114.7, 114.2, 109.6, 59.2, 26.7, 19.8.

IR (cm^{-1}): ṽ = 2920, 2851, 2554, 1687, 1583, 1229, 1143, 1018, 838, 735.

HRMS (ESI): calcd for $C_{26}H_{20}Cl_2N_3O_4^-$ $(M-H)^-$: 508.08364; found: 508.08377.

mp (uncorr.): 172.2-174.0 °C

References

(1) Borowiak, M.; Nahaboo, W.; Reynders, M.; Nekolla, K.; Jalinot, P.; Hasserodt, J.; Rehberg, M.; Delattre, M.; Zahler, S.; Vollmar, A.; et al. Photoswitchable Inhibitors of Microtubule Dynamics Optically Control Mitosis and Cell Death. *Cell* **2015**, *162* (2), 403–411. https://doi.org/10.1016/j.cell.2015.06.049.

(2) Merk, D.; Sreeramulu, S.; Kudlinzki, D.; Saxena, K.; Linhard, V.; Gande, S. L.; Hiller, F.; Lamers, C.; Nilsson, E.; Aagaard, A.; et al. Molecular Tuning of Farnesoid X Receptor Partial Agonism. *Nat. Commun.* **2019**, *10* (1), 1–14. https://doi.org/10.1038/s41467-019-10853-2.

(3) Gabler, M.; Kramer, J.; Schmidt, J.; Pollinger, J.; Weber, J.; Kaiser, A.; Löhr, F.; Proschak, E.; Schubert-Zsilavecz, M.; Merk, D. Allosteric Modulation of the Farnesoid X Receptor by a Small Molecule. *Sci. Rep.* **2018**, *8* (1), 1–11. https://doi.org/10.1038/s41598-018-25158-5.

(4) Akwabi-Ameyaw, A.; Bass, J. Y.; Caldwell, R. D.; Caravella, J. A.; Chen, L.; Creech, K. L.; Deaton, D. N.; Jones, S. A.; Kaldor, I.; Liu, Y.; et al. Conformationally Constrained Farnesoid X Receptor (FXR) Agonists: Naphthoic Acid-Based Analogs of GW 4064. *Bioorg. Med. Chem. Lett.* **2008**, *18* (15), 4339–4343. https://doi.org/10.1016/j.bmcl.2008.06.073.

(5) Leriche, G.; Budin, G.; Brino, L.; Wagner, A. Optimization of the Azobenzene Scaffold for Reductive Cleavage by Dithionite; Development of an Azobenzene Cleavable Linker for Proteomic Applications. *Eur. J. Org. Chem.* **2010**, *2010* (23), 4360–4364. https://doi.org/10.1002/ejoc.201000546.

(6) Maloney, P. R.; Parks, D. J.; Haffner, C. D.; Fivush, A. M.; Chandra, G.; Plunket, K. D.; Creech, K. L.; Moore, L. B.; Wilson, J. G.; Lewis, M. C.; et al. Identification of a Chemical Tool for the Orphan Nuclear Receptor FXR. *J. Med. Chem.* **2000**, *43* (16), 2971–2974. https://doi.org/10.1021/jm0002127.

^{1}H and ^{13}C NMR Spectra

3-((4-Hydroxyphenyl)diazenyl)benzoic acid (1)

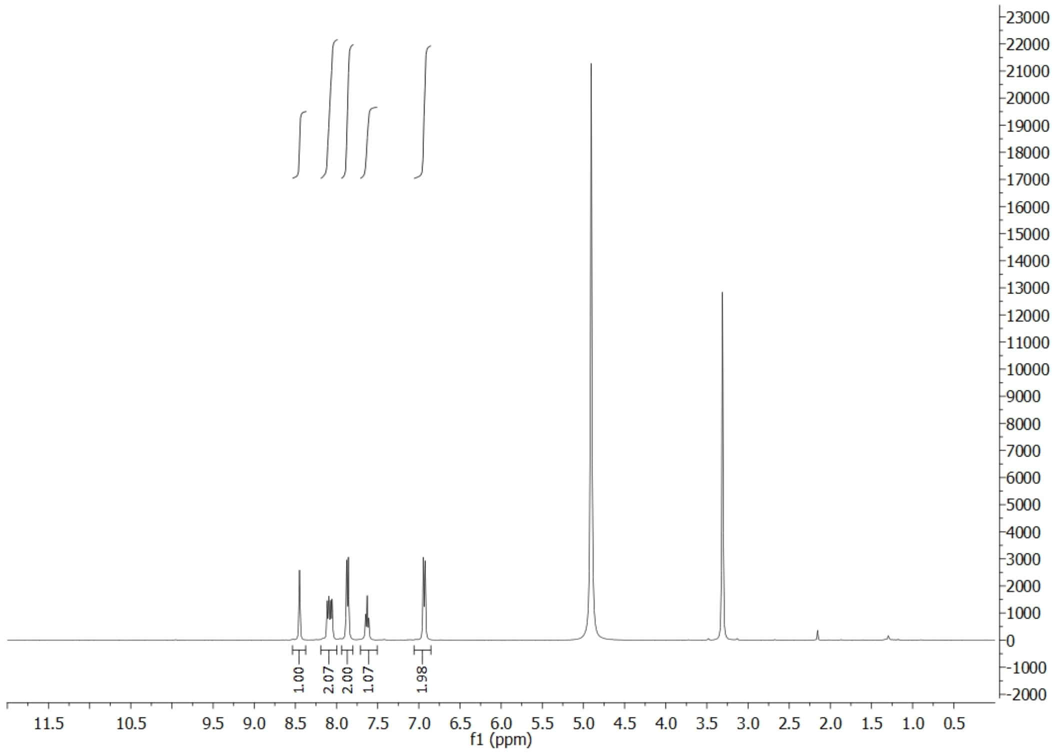

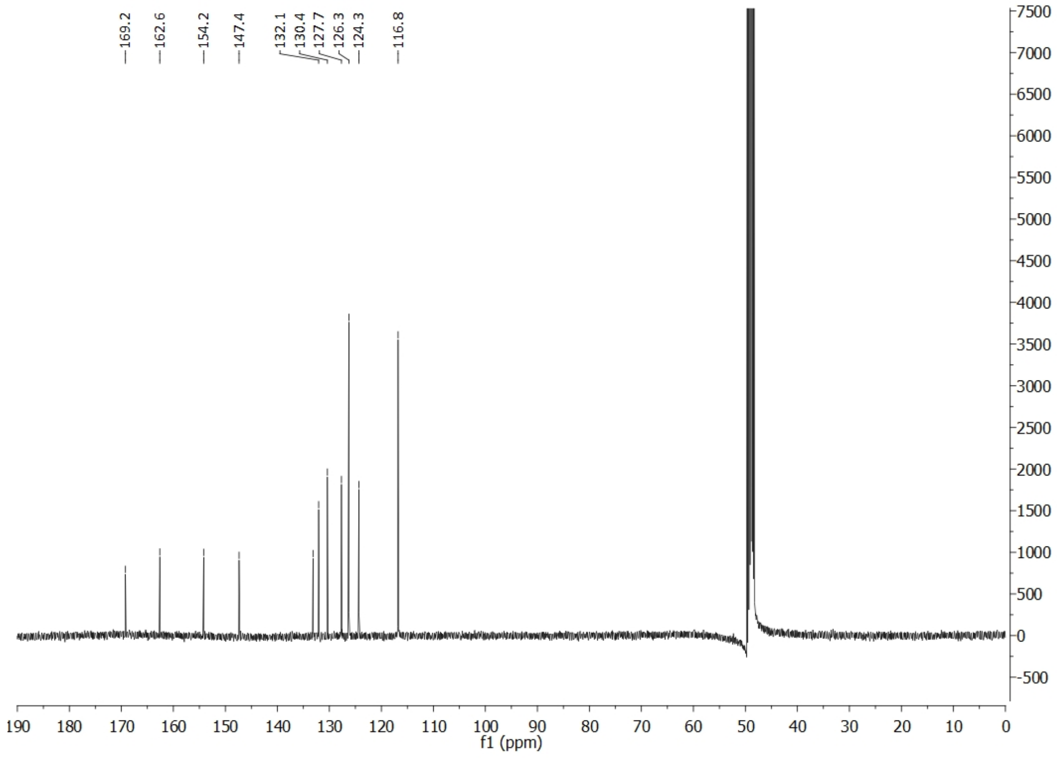

Methyl 3-((4-hydroxyphenyl)diazenyl)benzoate (2)

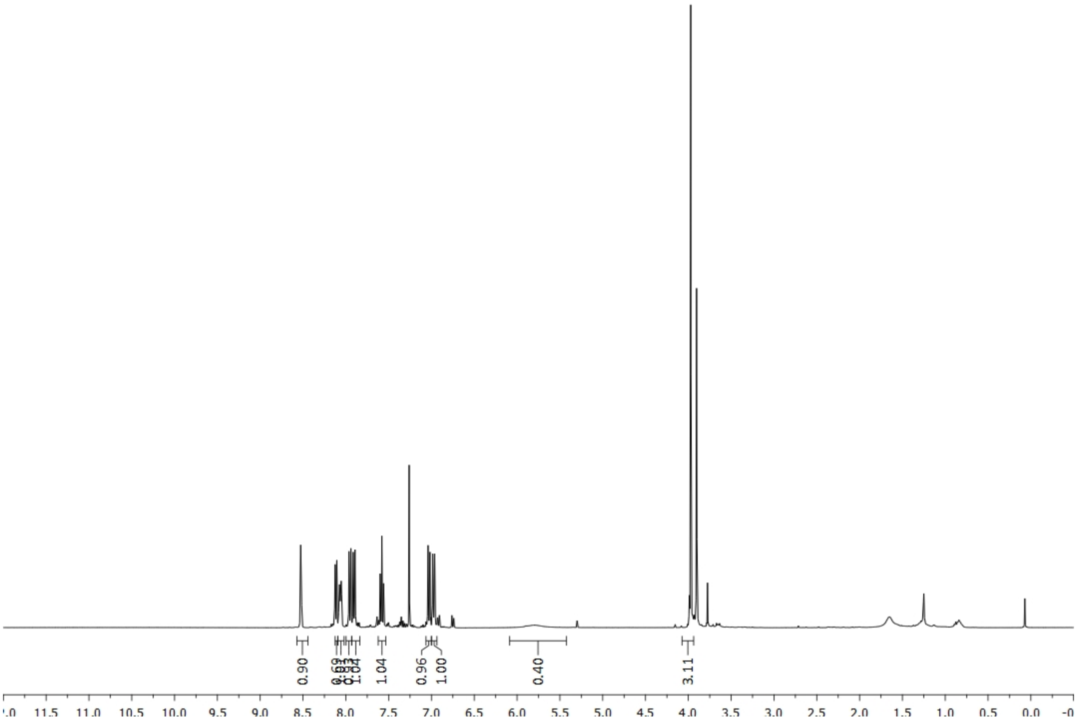

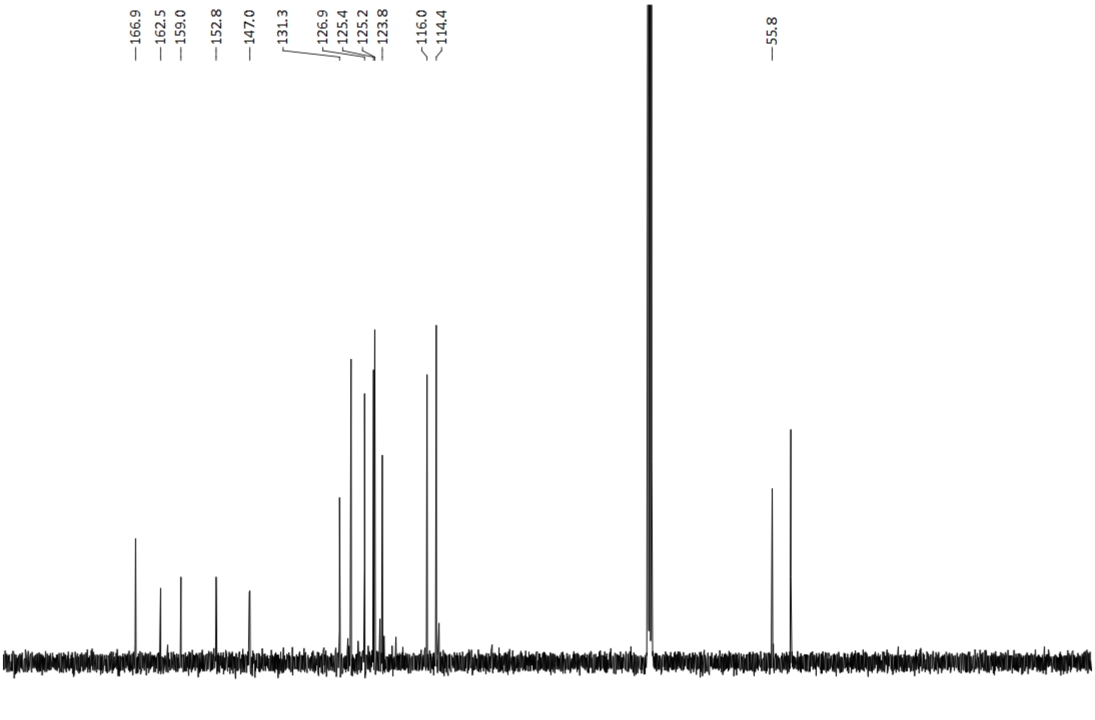

(*Z*)-2,6-Dichloro-*N*-hydroxybenzimidoyl chloride (3)

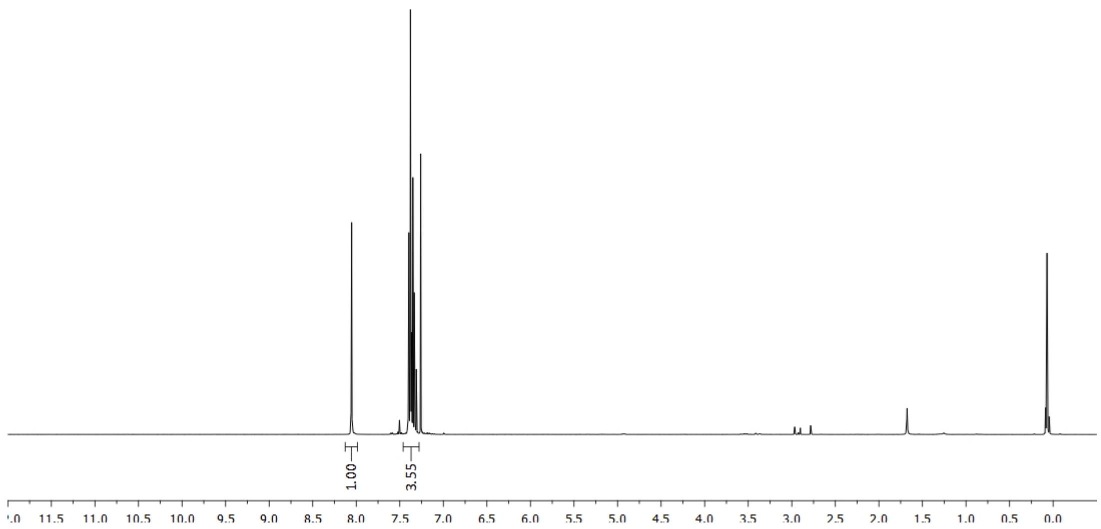

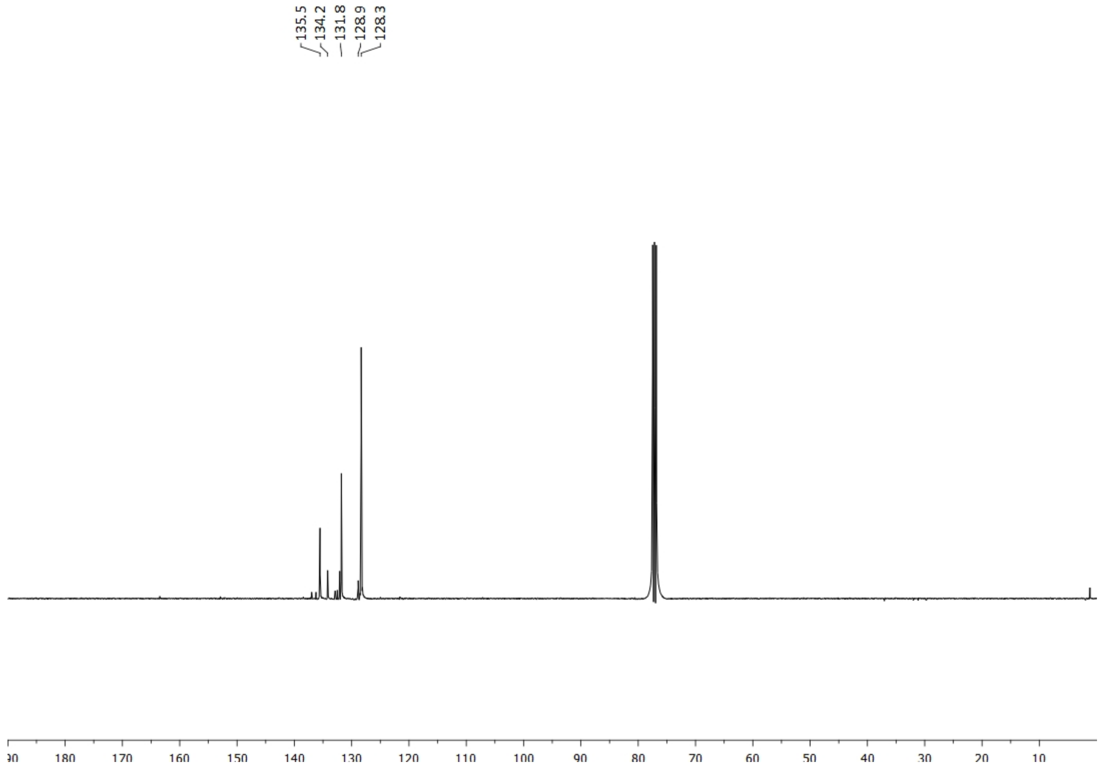

Methyl 3-(2,6-dichlorophenyl)-5-isopropylisoxazole-4-carboxylate (4)

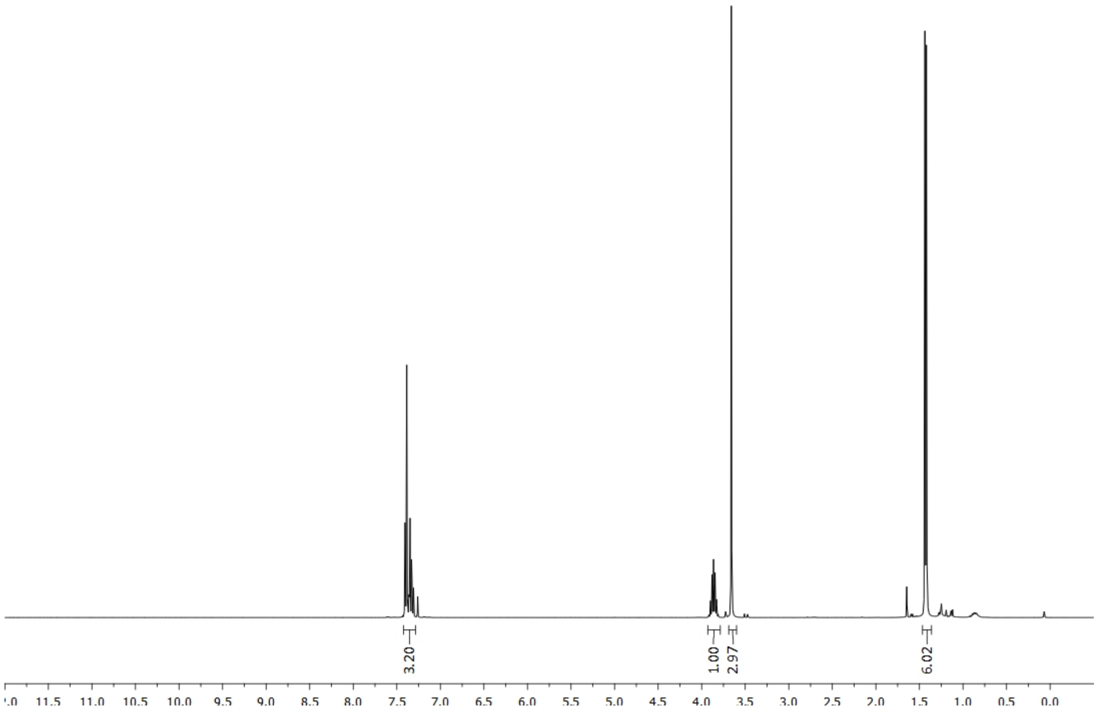

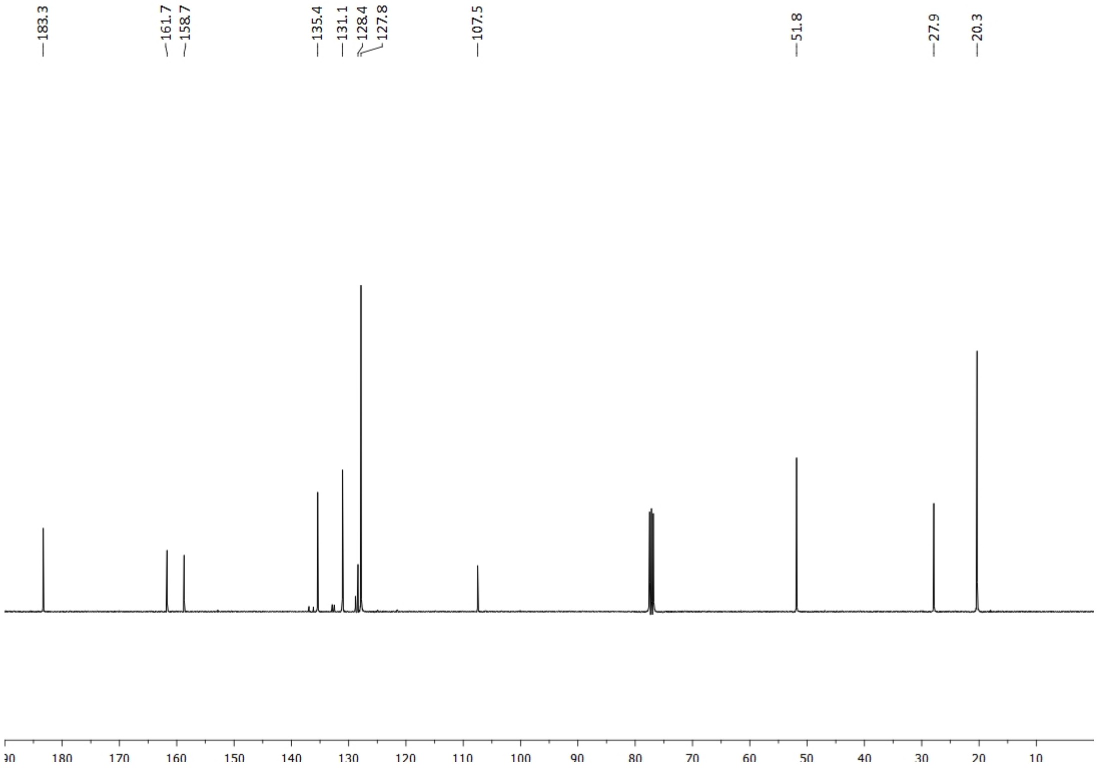

(3-(2,6-Dichlorophenyl)-5-isopropylisoxazol-4-yl)methanol (5)

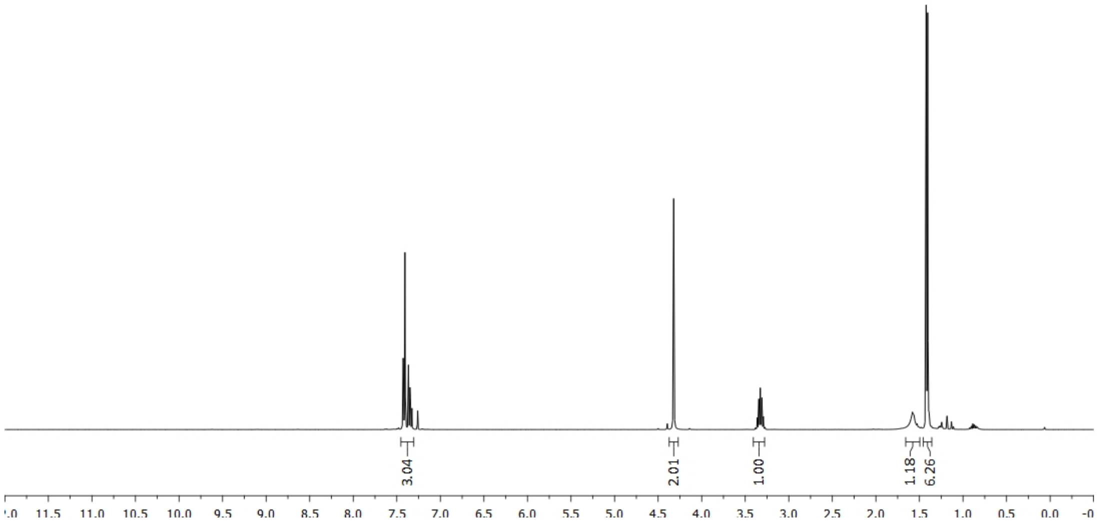

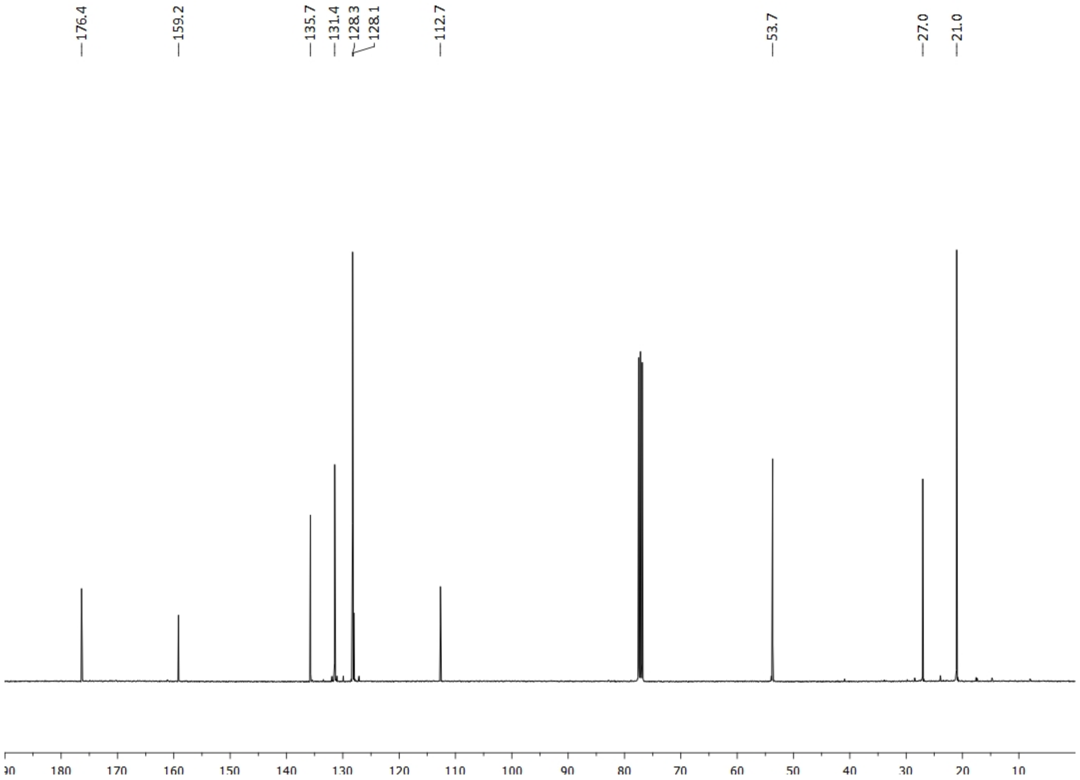

3-((4-((3-(2,6-dichlorophenyl)-5-isopropylisoxazol-4-yl)methoxy)phenyl)diazenyl)benzoic acid (AzoGW)

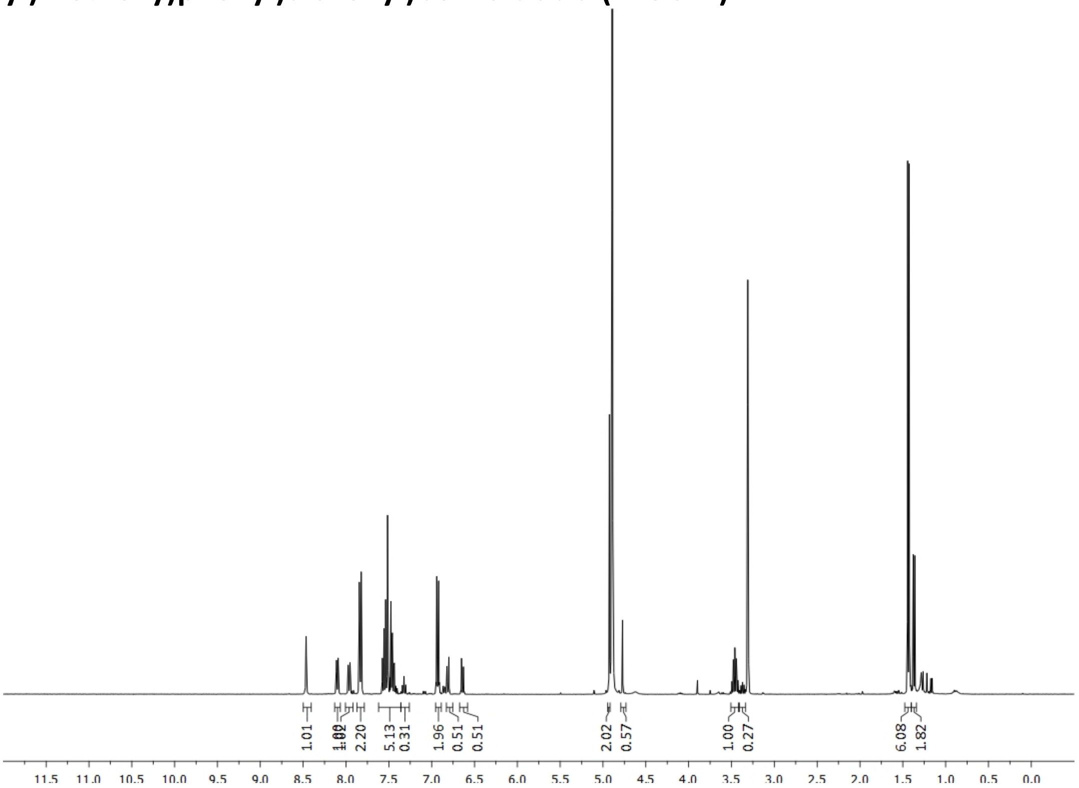

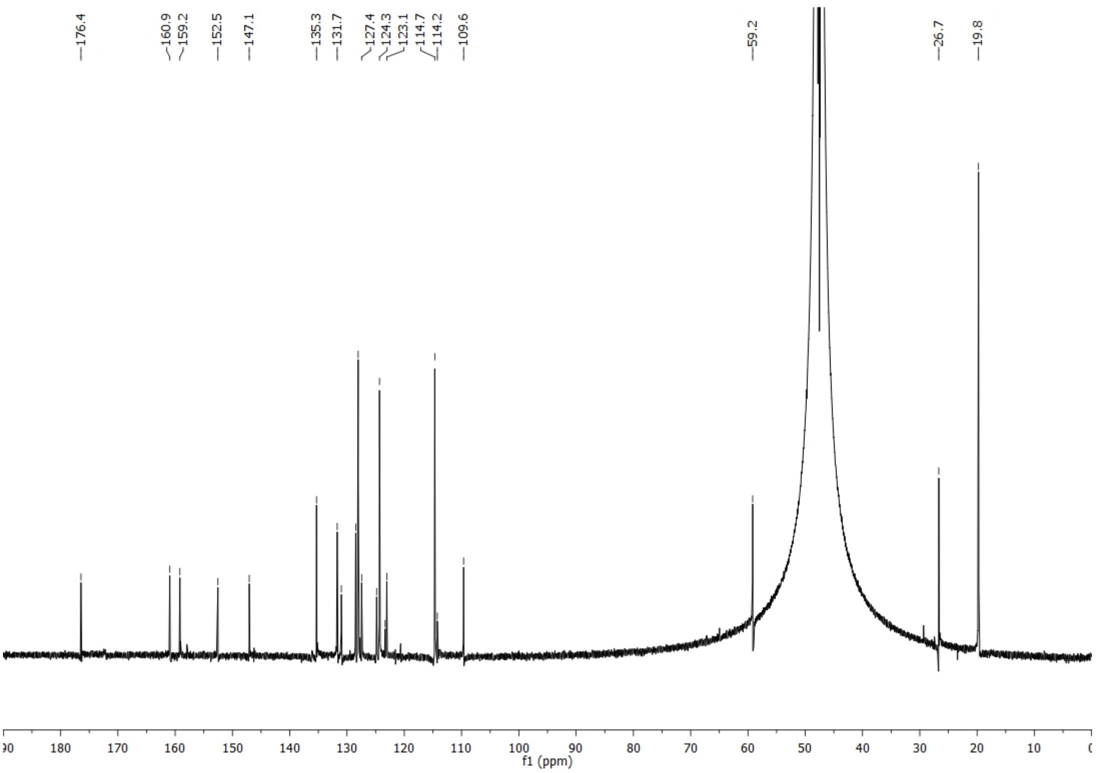

Supplement: Supplementary file 1 [file SC-011-C9SC02911G-s001.pdf]
